# Supplementary material for: Integrated Proteomics and Metabolomics Analysis Provides Insights into Ganoderic Acid Biosynthesis in Response to Methyl Jasmonate in Ganoderma Lucidum
Source: Int J Mol Sci. 2019 Dec 4;20(24):6116. doi: 10.3390/ijms20246116 (PMC6941157; doi:10.3390/ijms20246116)
Supplement: Supplementary file 1 [file ijms-20-06116-s001.zip › ijms-642095-revised-r1-supplementary/Table S1-S12/Table S1 DAPs M15 and M24 in common.docx]

Table S1. Differential abundance proteins of M15/C15 and M24/C24 in common

| **NO.** | **GL-ID** | **FC IN**  **M15/C15** | **FC IN**  **M24/C24** | **UniprotID** | **Gene name** | **Protein description** |
| --- | --- | --- | --- | --- | --- | --- |
| 1 | GL21210-R1_1 | 2.528 | 2.109 | Q06010 | STE23 | A-factor-processing enzyme |
| 2 | GL23892-R1_1 | 1.948 | 3.638 | P12866 | STE6 | Alpha-factor-transporting ATPase |
| 3 | GL24163-R1_1 | 2.188 | 2.197 | P38113 | ADH5 | Alcohol dehydrogenase 5 |
| 4 | GL22229-R1_1 | 7.821 | 0.578 | P00331 | ADH2 | Alcohol dehydrogenase 2 |
| 5 | GL24071-R2_1 | 3.377 | 9.439 | Q07747 | AAD4 | Probable aryl-alcohol dehydrogenase AAD4 |
| 6 | GL28863-R1_1 | 7.869 | 4.03 | P42884 | AAD14 | Putative aryl-alcohol dehydrogenase AAD14 |
| 7 | GL30395-R1_1 | 0.472 | 0.529 | P02407 | RPS17A | 40S ribosomal protein S17-A |
| 8 | GL25744-R1_1 | 0.277 | 0.358 | P0CX56 | RPS18B | 40S ribosomal protein S18-B |
| 9 | GL28146-R1_1 | 0.345 | 0.534 | P07281 | RPS19B | 40S ribosomal protein S19-B |
| 10 | GL22318-R2_1 | 0.274 | 0.449 | P0CX37 | RPS6A | 40S ribosomal protein S6-A |
| 11 | GL31415-R1_1 | 0.334 | 0.312 | Q02326 | RPL6A | 60S ribosomal protein L6-A |
| 12 | GL31410-R1_1 | 0.244 | 0.173 | P21951 | POL2 | DNA polymerase epsilon catalytic subunit A |
| 13 | GL23367-R1_1 | 0.444 | 0.329 | P15801 | MIP1 | DNA polymerase gamma |
| 14 | GL31536-R1_1 | 2.679 | 1.7 | P53819 | YRF1-6 | Y' element ATP-dependent helicase protein 1 copy 6 |
| 15 | GL26639-R1_1 | 6.018 | 0.428 | P20449 | DBP5 | ATP-dependent RNA helicase DBP5 |
| 16 | GL22129-R1_1 | 0.486 | 0.468 | P31115 | DEG1 | tRNA pseudouridine(38/39) synthase |
| 17 | GL25874-R1_1 | 3.345 | 8.316 | P53923 | NCS2 | Cytoplasmic tRNA 2-thiolation protein 2 |
| 18 | GL27820-R1_1 | 1.973 | 0.45 | P36421 | TYS1 | Tyrosine--tRNA ligase, cytoplasmic |
| 19 | GL19968-R1_1 | 2.686 | 23.496 | P22696 | ESS1 | Peptidyl-prolyl cis-trans isomerase ESS1 |
| 20 | GL23738-R1_1 | 2.43 | 5.338 | P07806 | VAS1 | Valine--tRNA ligase, mitochondrial |
| 21 | GL24443-R1_1 | 0.441 | 0.515 | P33892 | GCN1 | eIF-2-alpha kinase activator GCN1 |
| 22 | GL25322-R1_1 | 0.489 | 0.563 | Q12417 | PRP46 | Pre-mRNA-splicing factor PRP46 |
| 23 | GL16081-R1_1 | 2.354 | 1.812 | Q03677 | ADI1 | 1,2-dihydroxy-3-keto-5-methylthiopentene dioxygenase |
| 24 | GL31662-R1_1 | 0.331 | 0.34 | P05694 | MET6 | 5-methyltetrahydropteroyltriglutamate--homocysteine methyltransferase |
| 25 | GL19179-R1_1 | 0.291 | 0.44 | P18544 | ARG8 | Acetylornithine aminotransferase, mitochondrial |
| 26 | GL31667-R1_1 | 6.562 | 2.773 | Q00381 | APS2 | AP-2 complex subunit sigma |
| 27 | GL26084-R1_1 | 0.157 | 0.215 | P46682 | APL6 | AP-3 complex subunit beta |
| 28 | GL20491-R1_1 | 1.683 | 0.421 | P04807 | HXK2 | Hexokinase-2 |
| 29 | GL30114-R1_1 | 0.258 | 0.411 | P00924 | ENO1 | Enolase 1 |
| 30 | GL31363-R1_1 | 2.719 | 0.514 | P14065 | GCY1 | Glycerol 2-dehydrogenase (NADP(+) |
| 31 | GL22268-R1_1 | 1.762 | 0.514 | P00942 | TPI1 | Triosephosphate isomerase |
| 32 | GL17001-R1_1 | 0.405 | 0.448 | P35497 | SOR1 | Sorbitol dehydrogenase 1 |
| 33 | GL26490-R1_1 | 0.358 | 0.232 | P32891 | DLD1 | D-lactate dehydrogenase [cytochrome] 1, mitochondrial |
| 34 | GL21539-R1_1 | 10.608 | 2.444 | P28240 | ICL1 | Isocitrate lyase |
| 35 | GL28407-R1_1 | 0.328 | 0.361 | P08678 | CYR1 | Adenylate cyclase |
| 36 | GL20414-R1_1 | 3 | 1.697 | P07278 | BCY1 | cAMP-dependent protein kinase regulatory subunit |
| 37 | GL24512-R1_1 | 0.368 | 0.563 | P53909 | AAH1 | Adenine deaminase |
| 38 | GL16398-R1_1 | 1.901 | 5.202 | P38993 | FET3 | Iron transport multicopper oxidase FET3 |
| 39 | GL27379-R1_1 | 2.131 | 2.245 | P38169 | BNA4 | Kynurenine 3-monooxygenase |
| 40 | GL30169-R1_1 | 0.477 | 0.248 | Q10740 | LAP2 | Leukotriene A-4 hydrolase homolog |
| 41 | GL23263-R1_1 | 9.697 | 5.106 | Q05016 | YMR226C | NADP-dependent 3-hydroxy acid dehydrogenase |
| 42 | GL16520-R1_1 | 0.311 | 0.212 | Q12068 | GRE2 | NADPH-dependent methylglyoxal reductase GRE2 |
| 43 | GL18309-R1_1 | 2.932 | 3.242 | P53037 | PSD2 | Phosphatidylserine decarboxylase proenzyme 2 |
| 44 | GL19526-R1_1 | 1.999 | 2.052 | P39692 | MET10 | Sulfite reductase [NADPH] flavoprotein component |
| 45 | GL24313-R1_1 | 0.451 | 0.415 | P40083 | YER137C | Uncharacterized protein YEL137C |
| 46 | GL23646-R1_1 | 1.917 | 1.73 | Q12482 | AGC1 | Mitochondrial aspartate-glutamate transporter AGC1 |
| 47 | GL19075-R1_1 | 2.856 | 0.309 | P00431 | CCP1 | Cytochrome c peroxidase |
| 48 | GL24602-R1_1 | 4.786 | 2.423 | P32795 | YME1 | Mitochondrial inner membrane i-AAA protease supercomplex subunit YME1 |
| 49 | GL25065-R1_1 | 1.684 | 0.535 | P32573 | SPS19 | Peroxisomal 2,4-dienoyl-CoA reductase SPS19 |
| 50 | GL30526-R1_1 | 0.324 | 0.252 | P50264 | FMS1 | Polyamine oxidase FMS1 |
| 51 | GL22189-R1_1 | 0.223 | 0.313 | P06115 | CTT1 | Catalase T |
| 52 | GL27042-R1_1 | 3.233 | 5.427 | P54781 | ERG5 | Cytochrome P450 61 |
| 53 | GL22068-R1_1 | 1.787 | 7.275 | P08524 | ERG20 | Farnesyl pyrophosphate synthase |
| 54 | GL29873-R1_1 | 2.502 | 2.374 | P22146 | GAS1 | 1,3-beta-glucanosyltransferase GAS1 |
| 55 | GL20605-R1_1 | 0.316 | 0.334 | P32491 | MKK2 | MAP kinase kinase MKK2/SSP33 |
| 56 | GL24589-R1_1 | 0.473 | 0.357 | P14681 | KSS1 | Mitogen-activated protein kinase KSS1 |
| 57 | GL30014-R1_1 | 0.422 | 0.329 | P53886 | APC1 | Anaphase-promoting complex subunit 1 |
| 58 | GL30164-R1_1 | 1.791 | 0.529 | Q12440 | APC2 | Anaphase-promoting complex subunit 2 |
| 59 | GL27917-R1_1 | 0.528 | 0.437 | P48562 | CLA4 | Serine/threonine-protein kinase CLA4 |
