# Supplementary material for: Integrated Proteomics and Metabolomics Analysis Provides Insights into Ganoderic Acid Biosynthesis in Response to Methyl Jasmonate in Ganoderma Lucidum
Source: Int J Mol Sci. 2019 Dec 4;20(24):6116. doi: 10.3390/ijms20246116 (PMC6941157; doi:10.3390/ijms20246116)
Supplement: Supplementary file 1 [file ijms-20-06116-s001.zip › ijms-642095-revised-r1-supplementary/Table S1-S12/Table S6 GC-MS.docx]

Table S6 Changes of differential metabolites in response to MeJA obtained by GC-MS

| **NO.** | **Metabolites** | **Mass** | **r.t（min）** | **VIP** | **p value** | **FC(M24/C24)** |
| --- | --- | --- | --- | --- | --- | --- |
| 1 | DL-Anabasine | 156 | 17.38274615 | 1.18636 | 0.000168815 | 5082368.867 |
| 2 | Pipecolinic acid | 156 | 10.05971667 | 1.18633 | 0.00032445 | 3986418.434 |
| 3 | Norleucine | 158 | 17.60690667 | 1.18587 | 0.009182735 | 3504170.084 |
| 4 | norvaline | 144 | 11.45719286 | 1.18631 | 0.000366142 | 2192356.994 |
| 5 | N-methyltryptophan | 144 | 16.89806667 | 1.18613 | 0.002475361 | 2191184.419 |
| 6 | glutamine | 156 | 12.42138333 | 1.18613 | 0.002270313 | 1636894.365 |
| 7 | adrenaline | 116 | 6.281648571 | 1.18641 | 1.00726E-05 | 1566948.504 |
| 8 | N,N-dimethylarginine | 300 | 17.0222 | 1.18645 | 2.25703E-10 | 1523992.227 |
| 9 | Levoglucosan | 204 | 12.78901 | 1.18643 | 1.81341E-06 | 1331869.007 |
| 10 | 4-oxo-1H-quinoline-2-carboxylic acid | 318 | 11.4449 | 1.18636 | 9.53158E-05 | 972193.8056 |
| 11 | alpha-D-glucosamine 1-phosphate | 216 | 12.07346154 | 1.18636 | 9.48862E-05 | 947691.2185 |
| 12 | cycloleucine | 156 | 18.91153333 | 1.18638 | 5.09592E-05 | 778701.8521 |
| 13 | N-Methyl-L-glutamic acid | 244 | 10.25320909 | 1.18638 | 5.00508E-05 | 765930.1134 |
| 14 | O-Phosphoserine | 356 | 20.32635385 | 1.18629 | 0.000340089 | 611462.9154 |
| 15 | Sucrose-6-Phosphate | 315 | 27.55433077 | 1.1864 | 1.89636E-05 | 511879.4004 |
| 16 | alpha-Hydroxycholesterol | 298 | 6.882922 | 1.18646 | 6.42029E-07 | 448705.0758 |
| 17 | hypoxanthine | 280 | 12.92728 | 1.18642 | 3.089E-06 | 398663.6638 |
| 18 | Hexachlorobenzene | 286 | 16.46955 | 1.18646 | 5.50272E-05 | 386116.2965 |
| 19 | Allo-inositol | 318 | 11.18670769 | 1.18639 | 2.39339E-05 | 359437.8165 |
| 20 | thymine | 270 | 7.715180667 | 1.18631 | 0.000215793 | 275455.9384 |
| 21 | Glucosaminic acid | 288 | 15.79322 | 1.18639 | 2.20676E-05 | 257978.1756 |
| 22 | 2-hydroxy-3-isopropylbutanedioic acid | 275 | 10.8621 | 1.18637 | 5.59156E-05 | 246720.7642 |
| 23 | Cyclic AMP | 236 | 23.43349167 | 1.18586 | 0.004863617 | 224866.4289 |
| 24 | Diglycerol | 202 | 13.41775556 | 1.18556 | 0.010280123 | 197215.5741 |
| 25 | alanine | 262 | 19.03346 | 1.18646 | 1.09345E-08 | 170875.1611 |
| 26 | adenine | 264 | 20.7279 | 1.18647 | 4.94931E-06 | 157538.6206 |
| 27 | 2-Carboxybenzaldehyde | 236 | 15.08328333 | 1.1863 | 0.001547868 | 116028.2232 |
| 28 | Bis(2-hydroxypropyl)amine | 294 | 20.45232143 | 1.18631 | 0.000160871 | 111289.1291 |
| 29 | Thioctamide | 322 | 25.35303333 | 1.18626 | 0.0002138 | 32689.37684 |
| 30 | 2-Deoxy-D-galactose | 290 | 21.80098667 | 1.06276 | 0.006291748 | 158.7206686 |
| 31 | 3-hydroxy-L-proline | 158 | 12.28245714 | 1.02676 | 0.000570499 | 65.04828854 |
| 32 | 3'-adenylic acid | 192 | 22.6249 | 1.02818 | 0.000313647 | 35.56669797 |
| 33 | lysine | 174 | 14.74807333 | 1.00194 | 0.003275977 | 20.93760297 |
| 34 | D-erythro-sphingosine | 204 | 8.371416667 | 1.15843 | 0.000585728 | 7.114837222 |
| 35 | phytosphingosine | 204 | 6.472286 | 1.14931 | 0.020644348 | 6.441220353 |
| 36 | 3,5-Dihydroxyphenylglycine | 318 | 11.28892222 | 1.15883 | 5.79357E-05 | 6.403917249 |
| 37 | D-alanyl-D-alanine | 116 | 5.219412 | 1.10449 | 0.000530269 | 5.999013928 |
| 38 | 2'-deoxyguanosine | 214 | 25.41165882 | 1.15543 | 0.016374969 | 5.3029859 |
| 39 | guanosine | 296 | 24.42798889 | 1.14617 | 0.018962099 | 4.499876592 |
| 40 | 2-Amino-3-methyl-1-butanol | 144 | 20.60195 | 1.16044 | 0.009106971 | 4.178114356 |
| 41 | Cysteinylglycine | 257 | 11.02485625 | 1.15856 | 0.000568712 | 4.148606176 |
| 42 | guanine | 352 | 17.98645294 | 1.15097 | 0.000966719 | 4.045465709 |
| 43 | glucose-6-phosphate | 218 | 23.23881176 | 1.15266 | 0.005493057 | 3.846200028 |
| 44 | 2-amino-2-methylpropane-1,3-diol | 218 | 8.008707059 | 1.15574 | 0.005545363 | 3.776566854 |
| 45 | glycine | 102 | 5.251401176 | 1.12607 | 0.00070627 | 3.678486602 |
| 46 | Lumazine | 308 | 22.3241875 | 1.08164 | 0.004055213 | 3.293268522 |
| 47 | serine | 204 | 7.034217222 | 1.1374 | 0.000760841 | 2.805491451 |
| 48 | phenylalanine | 218 | 10.40336667 | 1.16333 | 0.000765671 | 2.788554615 |
| 49 | Cytosin | 254 | 9.079653889 | 1.14881 | 0.000527255 | 2.682449 |
| 50 | O-Succinylhomoserine | 201 | 10.03385882 | 1.11737 | 0.020807419 | 2.484843394 |
| 51 | glycerol | 256 | 9.725748125 | 1.12681 | 0.002034059 | 2.476646304 |
| 52 | 1-Monopalmitin | 174 | 23.54863889 | 1.12504 | 0.002435873 | 2.324017376 |
| 53 | uracil | 241 | 6.787513333 | 1.16532 | 0.000286116 | 2.234839528 |
| 54 | adenosine | 322 | 23.58812222 | 1.13079 | 0.015138421 | 2.233060103 |
| 55 | 2,6-Diaminopimelic acid | 201 | 10.11052353 | 1.10856 | 0.029303575 | 2.225244284 |
| 56 | 5-Aminoimidazole-4-carboxamide | 311 | 11.36135294 | 1.01285 | 0.02706055 | 1.943259152 |
| 57 | urea | 189 | 5.617107059 | 1.18428 | 7.30038E-06 | 1.915554729 |
| 58 | 5-Methoxyindole-3-acetic acid | 260 | 20.3926 | 1.11477 | 0.02069664 | 1.892913451 |
| 59 | 2,4-diaminobutyric acid | 229 | 10.42521875 | 1.16813 | 0.000654408 | 1.860190638 |
| 60 | Sedoheptulose | 333 | 14.89981429 | 1.15876 | 0.002631293 | 1.82959003 |
| 61 | 2-methylfumarate | 255 | 7.561414706 | 1.18158 | 2.61665E-05 | 1.795941692 |
| 62 | 3-hydroxypyruvate | 58 | 6.040026 | 1.17379 | 0.000346286 | 1.746189746 |
| 63 | alpha-Aminoadipic acid | 154 | 10.44334706 | 1.15375 | 0.001962335 | 1.733523382 |
| 64 | proline | 142 | 6.320711111 | 1.13487 | 0.003403633 | 1.724368883 |
| 65 | threonine | 294 | 7.352162222 | 1.15344 | 0.001206242 | 1.680599536 |
| 66 | Lyxonic acid, 1,4-lactone | 275 | 10.47784444 | 1.14553 | 0.004181773 | 1.660722471 |
| 67 | Adenosine 5-monophosphate | 315 | 27.86601765 | 1.15717 | 0.001136483 | 1.652542831 |
| 68 | 8-Aminocaprylic acid | 174 | 15.04982941 | 1.17858 | 0.000239871 | 1.637981776 |
| 69 | N-Acetyltryptophan | 202 | 12.53035 | 1.15544 | 0.002725907 | 1.634982702 |
| 70 | gluconic acid | 334 | 15.92644444 | 1.11949 | 0.007397075 | 1.626226349 |
| 71 | trans,trans-Muconic acid | 271 | 6.025955882 | 1.1619 | 0.00096888 | 1.598465323 |
| 72 | L-cysteine | 220 | 9.464541667 | 1.12525 | 0.004943832 | 1.539896647 |
| 73 | aspartic acid | 232 | 9.013959444 | 1.05379 | 0.040846862 | 1.531168018 |
| 74 | maltose | 362 | 15.25951111 | 1.16414 | 0.001772569 | 1.50977466 |
| 75 | Isoleucine | 158 | 6.228268333 | 1.16189 | 0.000974488 | 1.457273339 |
| 76 | Perillyl Alcohol | 181 | 16.56573889 | 1.18421 | 1.22776E-05 | 1.455783393 |
| 77 | glutamic acid | 246 | 10.27518889 | 1.05457 | 0.039661658 | 1.454843222 |
| 78 | N-Acetyl-D-galactosamine | 320 | 17.20749444 | 1.16809 | 0.000662376 | 1.393565741 |
| 79 | 2-Indanone | 215 | 11.86688889 | 1.15015 | 0.001988836 | 1.381136147 |
| 80 | valine | 144 | 5.286631667 | 1.07848 | 0.021143223 | 1.361257204 |
| 81 | o-Hydroxyhippuric acid | 411 | 26.83527647 | 1.17697 | 0.000128039 | 1.355497058 |
| 82 | tyrosine | 226 | 12.35172222 | 1.17686 | 0.000150073 | 1.342346644 |
| 83 | phenylethylamine | 174 | 9.66842 | 1.13386 | 0.008637284 | 1.341152191 |
| 84 | 1-Hydroxy-2-naphthoic acid | 317 | 16.79642353 | 1.15492 | 0.002283822 | 1.337789584 |
| 85 | Corticosterone | 251 | 29.81162778 | 1.0819 | 0.02820055 | 1.334143209 |
| 86 | Guanidinosuccinic acid | 343 | 9.916125625 | 1.18567 | 2.43788E-06 | 1.320723227 |
| 87 | 5-hydroxytryptophan | 290 | 17.92326875 | 1.12509 | 0.004991316 | 1.30075477 |
| 88 | D-galacturonic acid | 333 | 15.14904118 | 1.1489 | 0.004369526 | 1.288927251 |
| 89 | Glucose-1-phosphate | 217 | 8.947292222 | 1.18523 | 2.97287E-06 | 1.27118069 |
| 90 | sulfuric acid | 226 | 6.053130625 | 1.15003 | 0.002779064 | 1.270470714 |
| 91 | sorbitol | 305 | 21.51961765 | 1.1405 | 0.00580182 | 1.247143545 |
| 92 | 6-deoxy-D-glucose | 322 | 11.69385556 | 1.17743 | 0.000240324 | 1.22991932 |
| 93 | ribitol | 217 | 15.81011667 | 1.05072 | 0.020825832 | 1.229514408 |
| 94 | xylitol | 307 | 11.40502222 | 1.16671 | 0.000424439 | 1.217326977 |
| 95 | Zymosterol | 255 | 32.02945 | 1.05011 | 0.036870947 | 1.194471173 |
| 96 | oxalic acid | 190 | 7.923878889 | 1.16623 | 0.001188528 | 1.19312422 |
| 97 | hydrocinnamic acid | 276 | 10.674875 | 1.17969 | 7.60849E-05 | 1.154373212 |
| 98 | Sophorose | 204 | 16.40002778 | 1.12985 | 0.006875944 | 1.15135044 |
| 99 | phosphate | 314 | 5.943169444 | 1.16157 | 0.001776224 | 1.149933383 |
| 100 | Carbazole | 214 | 6.843865 | 1.05685 | 0.034755475 | 1.144072968 |
| 101 | fucose | 322 | 11.88845556 | 1.10417 | 0.020122631 | 1.135820558 |
| 102 | Gluconic lactone | 117 | 11.09689167 | 1.15964 | 0.0021689 | 1.122473388 |
| 103 | 1-Kestose | 361 | 33.91663333 | 1.09902 | 0.015611591 | 1.1055468 |
| 104 | oxoproline | 156 | 6.729215 | 1.16917 | 0.00093046 | 1.09863949 |
| 105 | beta-Mannosylglycerate | 216 | 22.42303333 | 1.09511 | 0.023296954 | 1.093988709 |
| 106 | Lyxose | 174 | 22.03126667 | 1.1135 | 0.006625646 | 1.087597632 |
| 107 | trans-4-hydroxy-L-proline | 146 | 14.52951538 | 1.13594 | 0.008212333 | 1.073542639 |
| 108 | D-Talose | 321 | 14.62914444 | 1.15135 | 0.002390203 | 1.073379503 |
| 109 | galactose | 157 | 11.03817222 | 1.02906 | 0.04816935 | 1.068210499 |
| 110 | dihydrotestosterone | 226 | 11.08030556 | 1.15096 | 0.001280558 | 1.048923063 |
| 111 | N-Acetyl-beta-D-mannosamine | 373 | 20.18328333 | 1.03048 | 0.03148524 | 1.036873967 |
| 112 | Ergosterol | 363 | 29.94891176 | 1.06901 | 0.046448516 | 1.025107166 |
| 113 | 4-Vinylphenol dimer | 192 | 5.70689 | 1.07005 | 0.037374257 | 0.977546249 |
| 114 | fumaric acid | 245 | 6.943161111 | 1.0665 | 0.022828161 | 0.968612513 |
| 115 | Digalacturonic acid | 363 | 21.84871667 | 1.14051 | 0.002296416 | 0.929505467 |
| 116 | sucrose | 361 | 23.88349444 | 1.13168 | 0.007398049 | 0.923322808 |
| 117 | 1,3-diaminopropane | 174 | 8.30794 | 1.13351 | 0.008196381 | 0.911798801 |
| 118 | 2-Deoxyerythritol | 117 | 6.150842941 | 1.03266 | 0.024952574 | 0.910842433 |
| 119 | 5-Methoxytryptamine | 174 | 6.085878125 | 1.13096 | 0.011424846 | 0.906256588 |
| 120 | palmitic acid | 313 | 16.78006111 | 1.08297 | 0.013128438 | 0.903739756 |
| 121 | Glutaconic acid | 215 | 10.21834444 | 1.11041 | 0.017626997 | 0.886813738 |
| 122 | trehalose | 363 | 24.67315882 | 1.16073 | 0.002562563 | 0.883138839 |
| 123 | 5-Aminovaleric acid | 174 | 7.84391 | 1.05273 | 0.038540625 | 0.877127698 |
| 124 | fructose-6-phosphate | 315 | 20.92511111 | 1.13503 | 0.003402398 | 0.87051488 |
| 125 | 2-Amino-1-phenylethanol | 174 | 7.541763333 | 1.06514 | 0.02060996 | 0.864613714 |
| 126 | 3-phenylcatechol | 330 | 20.663575 | 1.16663 | 0.000423162 | 0.86012842 |
| 127 | 11-beta-prostaglandin-F-2-alpha | 207 | 5.164806111 | 1.11164 | 0.004823806 | 0.855334542 |
| 128 | myo-inositol | 305 | 17.41299444 | 1.14475 | 0.002390198 | 0.854584301 |
| 129 | mannitol | 322 | 14.82193889 | 1.17334 | 0.000726128 | 0.840759556 |
| 130 | N-(2-hydroxyethyl)-iminodiacetic acid | 359 | 9.201893889 | 1.1061 | 0.007410876 | 0.823057765 |
| 131 | L-Malic acid | 335 | 8.616317778 | 1.14338 | 0.002049288 | 0.823027552 |
| 132 | creatine degr | 147 | 5.274990556 | 1.15503 | 0.002701986 | 0.815143176 |
| 133 | 3,4-Dihydroxyphenylglycol | 355 | 6.95124875 | 1.15268 | 0.004645231 | 0.79320292 |
| 134 | 3-Hydroxypropionic acid | 103 | 7.677492941 | 1.1794 | 5.12988E-05 | 0.782632901 |
| 135 | ribulose-5-phosphate | 357 | 17.6230875 | 1.08783 | 0.013488776 | 0.756993538 |
| 136 | alpha-ketoglutaric acid | 288 | 9.696136667 | 1.14397 | 0.001873521 | 0.714156286 |
| 137 | stearic acid | 341 | 20.00242778 | 1.17543 | 8.82913E-05 | 0.691004959 |
| 138 | Pelargonic acid | 215 | 7.102268333 | 1.14753 | 0.007448769 | 0.681215317 |
| 139 | prostaglandin A2 | 119 | 29.21979444 | 1.13217 | 0.006669551 | 0.66714903 |
| 140 | beta-Glycerophosphoric acid | 238 | 14.88222778 | 1.09651 | 0.004603238 | 0.642691931 |
| 141 | 3-Phenyllactic acid | 325 | 26.85007222 | 1.16047 | 0.003230406 | 0.639706114 |
| 142 | glutathione | 213 | 18.67203333 | 1.11146 | 0.019215109 | 0.601566324 |
| 143 | 4-hydroxybutyrate | 117 | 7.257083333 | 1.10689 | 0.035225807 | 0.59966568 |
| 144 | Pyruvic acid | 251 | 23.27866667 | 1.08726 | 0.020574309 | 0.521763653 |
| 145 | pyrogallol | 239 | 6.870180769 | 1.14005 | 0.002218556 | 0.517612584 |
| 146 | Maleimide | 154 | 10.34459286 | 1.16582 | 0.003036459 | 0.450269682 |
| 147 | Threitol | 84 | 8.820332778 | 1.17617 | 0.001610458 | 0.316903197 |
| 148 | tetracosane | 57 | 19.34785556 | 1.16428 | 0.001579533 | 0.285242278 |
| 149 | L-glutamic acid | 84 | 9.183895294 | 1.17926 | 0.001098074 | 0.265058809 |
| 150 | 5-Methylresorcinol | 268 | 9.517901429 | 1.18646 | 1.52242E-06 | 1.17347E-05 |
| 151 | 2-mercaptoethanesulfonic acid | 214 | 12.808 | 1.18644 | 0.000162897 | 3.26884E-06 |
| 152 | 3-hydroxybutyric acid | 318 | 14.43385 | 1.18646 | 1.51537E-08 | 1.65253E-06 |
| 153 | N-Acetyl-5-hydroxytryptamine | 188 | 21.79218333 | 1.18645 | 1.91858E-08 | 1.02283E-06 |
| 154 | Gentiobiose | 204 | 25.28212727 | 1.18613 | 0.01102586 | 1.03883E-08 |
