# Supplementary material for: Integrated Proteomics and Metabolomics Analysis Provides Insights into Ganoderic Acid Biosynthesis in Response to Methyl Jasmonate in Ganoderma Lucidum
Source: Int J Mol Sci. 2019 Dec 4;20(24):6116. doi: 10.3390/ijms20246116 (PMC6941157; doi:10.3390/ijms20246116)
Supplement: Supplementary file 1 [file ijms-20-06116-s001.zip › ijms-642095-revised-r1-supplementary/Table S1-S12/Table S7 LC-MS.docx]

Table S7 Changes of differential metabolites in response to MeJA obtained by LC-MS

| NO | A.m | m/z | r.t(min) | Metabolites | p value | FC |
| --- | --- | --- | --- | --- | --- | --- |
| 1 | neg | 395.3886476 | 21.30974764 | Hexacosanoic acid | 0.011517562 | 3.12565441 |
| 2 | neg | 381.3718862 | 20.86205992 | Lepidiumsesterterpenol | 0.016297433 | 2.699044253 |
| 3 | neg | 399.3265735 | 19.60807387 | (24R)-Cholest-5-ene-3-beta,7-alpha,24-triol | 0.02652294 | 2.68713569 |
| 4 | pos | 300.0928455 | 0.79344987 | 8-Hydroxyguanosine | 0.028025659 | 2.499580138 |
| 5 | pos | 314.1095341 | 0.760484345 | Amoxapine | 0.026080887 | 2.474638311 |
| 6 | neg | 397.3688507 | 20.24774178 | 2-Hydroxy-22-methyltetracosanoic acid | 0.0357413 | 2.396420978 |
| 7 | pos | 260.0970115 | 0.706772024 | Glutamyl-Hydroxyproline | 0.007974495 | 2.124623169 |
| 8 | pos | 274.1146776 | 0.721720561 | 4a-peroxy-tetrahydrobiopterin | 0.020570682 | 1.99976584 |
| 9 | neg | 253.2520945 | 19.7425979 | 2-Heptadecanone | 0.004218065 | 1.990660538 |
| 10 | neg | 383.3524962 | 19.93175496 | Cerebronic acid | 0.031364711 | 1.830525612 |
| 11 | neg | 367.3572518 | 20.4663059 | Tetracosanoic acid | 0.029967405 | 1.830389465 |
| 12 | neg | 393.3727082 | 20.54919357 | 7,9-Hexacosanedione | 0.016537615 | 1.822505984 |
| 13 | neg | 353.34146 | 20.10758013 | 6-Hydroxy-8-tricosanone | 0.021697828 | 1.80843691 |
| 14 | pos | 113.0347105 | 0.813599362 | Uracil | 0.018751083 | 1.778475509 |
| 15 | neg | 281.2481584 | 19.74380727 | (Z)-13-Octadecenoic acid | 0.004526951 | 1.763022576 |
| 16 | neg | 459.3105145 | 19.25508004 | Tetrahydroxyergosta-7,22-dien-6-one | 0.014261584 | 1.728679268 |
| 17 | neg | 257.1500182 | 5.235181282 | Flavidulol A | 0.029846386 | 1.725118493 |
| 18 | pos | 152.0521977 | 0.735481051 | 2,5-Dihydro-2,4,5-trimethylthiazole | 0.016365266 | 1.647699345 |
| 19 | neg | 355.3205884 | 19.36880076 | 2(R)-hydroxydocosanoic acid | 0.019495018 | 1.491712861 |
| 20 | neg | 331.2275268 | 18.35546809 | 16-a-Hydroxypregnenolone | 0.005964964 | 1.465008313 |
| 21 | pos | 284.0979845 | 0.755667011 | Guanosine | 0.023356706 | 1.461812796 |
| 22 | neg | 283.2641707 | 19.74626763 | Stearic acid | 0.007994008 | 1.453246766 |
| 23 | neg | 134.0472816 | 3.623823042 | Adenine | 0.015181765 | 1.420302298 |
| 24 | pos | 298.1135067 | 0.761417663 | 1-Methylguanosine | 0.013096397 | 1.419790766 |
| 25 | neg | 229.1555344 | 7.455185111 | Isoleucyl-Valine | 0.028271687 | 1.357689282 |
| 26 | neg | 339.3257552 | 19.79487865 | Behenic acid | 0.046193285 | 1.356431064 |
| 27 | pos | 395.3302309 | 18.69469376 | Ergosta-4,6,8(14),22-tetraen-3-ol | 0.008421302 | 1.352054787 |
| 28 | neg | 314.2075787 | 8.72445437 | 3-Oxotetradecanoic acid glycerides | 0.045139744 | 1.344298691 |
| 29 | pos | 231.1367087 | 7.795829456 | Furanodienone | 0.003303974 | 1.323241004 |
| 30 | neg | 445.333667 | 19.11278374 | 3,5,9-Trihydroxyergost-7-en-6-one | 0.017359477 | 1.305724592 |
| 31 | pos | 310.3087714 | 15.83101959 | N-Hexadecanoylpyrrolidine | 0.041589445 | 1.233273579 |
| 32 | pos | 315.2308774 | 19.3337541 | 4-Nerolidylcatechol | 0.040604824 | 1.226077295 |
| 33 | neg | 313.2171443 | 19.17289097 | 4-Nerolidylcatechol | 0.017014643 | 1.22431844 |
| 34 | pos | 328.3199691 | 15.83101959 | Stearoylethanolamide | 0.045440808 | 1.224215199 |
| 35 | neg | 299.2592934 | 18.10008145 | (R)-3-Hydroxy-Octadecanoic acid | 0.015967833 | 1.202850994 |
| 36 | neg | 341.1090812 | 0.707953533 | Neotrehalose | 0.000957654 | 1.096176636 |
| 37 | neg | 255.2325849 | 17.76040957 | Palmitic acid | 0.04083537 | 0.945576952 |
| 38 | pos | 111.0199591 | 29.70044566 | 2,2-Dimethyloxirane | 0.03470582 | 0.908570945 |
| 39 | neg | 241.2172613 | 17.28768253 | 13-Methylmyristic acid | 0.036254924 | 0.898617745 |
| 40 | pos | 200.0460862 | 2.872886627 | Tryptophanol | 0.024425945 | 0.862148302 |
| 41 | neg | 227.2017508 | 16.74080459 | Myristic acid | 0.011454579 | 0.85804883 |
| 42 | pos | 156.0379612 | 2.872886627 | (R)-2,3-Dihydroxy-3-methylbutanoate | 0.024388802 | 0.855521548 |
| 43 | pos | 154.041421 | 2.872886627 | Caproate (6:0) | 0.026465449 | 0.852576419 |
| 44 | neg | 181.0716568 | 0.695876682 | L-Lysine | 0.003595883 | 0.848564402 |
| 45 | pos | 148.0476861 | 0.752799116 | 5-Methylcytosine | 0.00783033 | 0.774604778 |
| 46 | pos | 295.2251046 | 13.86897133 | (R)-3-Hydroxy-hexadecanoic acid | 0.03039431 | 0.755379395 |
| 47 | neg | 311.2225029 | 20.0675216 | 13-L-Hydroperoxylinoleic acid | 0.005316854 | 0.721781202 |
| 48 | pos | 295.2251046 | 16.44393831 | 15,16-Epoxy-9,12-octadecadienoic acid | 0.025079265 | 0.712186149 |
| 49 | neg | 311.2213237 | 14.26972132 | 5,8,12-Trihydroxy-9-octadecenoic acid | 0.011581596 | 0.709207407 |
| 50 | pos | 277.2149762 | 16.4428526 | (E)-11-Hexadecenoic acid | 0.030009415 | 0.699331048 |
| 51 | pos | 317.206909 | 16.4509445 | 6-Hydroxy-9,12,14-octadecatrienoic acid | 0.043728232 | 0.675512418 |
| 52 | pos | 293.2094996 | 22.63593898 | 3-Oxohexadecanoic acid | 0.016379919 | 0.674779588 |
| 53 | neg | 157.1234274 | 20.14215706 | 2-Methylpropyl 3-methylbutanoate | 0.025877267 | 0.674346694 |
| 54 | neg | 242.177428 | 20.13347929 | N-Undecanoylglycine | 0.043317242 | 0.660774238 |
| 55 | neg | 295.2272337 | 15.50242678 | (Z)-13-Oxo-9-octadecenoic acid | 0.002257027 | 0.642707221 |
| 56 | pos | 295.2251046 | 24.45424849 | 3-hydroxyhexadecanoic acid | 0.000145882 | 0.606472327 |
| 57 | neg | 311.2213237 | 13.62036642 | 5,8,12-Trihydroxy-9-octadecenoic acid | 0.00326758 | 0.601342657 |
| 58 | pos | 277.2150118 | 24.45034202 | (Z)-13-Hexadecenoic acid | 0.000169236 | 0.591813097 |
| 59 | neg | 293.211509 | 15.48780053 | 9-Oxoode | 0.023881341 | 0.581576009 |
| 60 | pos | 575.4765958 | 24.38240897 | DG(15:0/18:4(6Z,9Z,12Z,15Z)/0:0) | 0.000316057 | 0.580779698 |
| 61 | pos | 597.4442967 | 24.45424849 | 1,2-Epoxy-octahydro-psi,psi-carotene | 0.000137594 | 0.573942899 |
| 62 | neg | 242.1785169 | 10.63526221 | N-Undecanoylglycine | 0.013743686 | 0.526299188 |
| 63 | neg | 573.4495807 | 20.0204046 | DG(15:0/18:4(6Z,9Z,12Z,15Z)/0:0) | 0.000752889 | 0.518393168 |
| 64 | neg | 277.2173571 | 15.49872307 | Linolenelaidic acid | 0.006482618 | 0.507079916 |
| 65 | pos | 330.0617287 | 0.754321668 | 2',3'-cyclic AMP | 0.011880118 | 0.411803976 |
| 66 | pos | 308.0931374 | 0.755364122 | Glutathione | 0.006598036 | 0.358437958 |
| 67 | pos | 611.4658288 | 25.62087594 | DG(14:1/22:6) | 0.041218026 | 0.312573021 |
| 68 | neg | 311.2213253 | 15.48844744 | 13-L-Hydroperoxylinoleic acid | 0.021149325 | 0.283831937 |
| 69 | pos | 520.3364152 | 15.50608272 | LysoPC(18:2(9Z,12Z)) | 0.042315869 | 0.271516307 |
| 70 | neg | 343.2473615 | 13.01708185 | Dihomo-linoleate (20:2n6) | 0.045675709 | 0.175177196 |
