# Supplementary material for: Integrated Proteomics and Metabolomics Analysis Provides Insights into Ganoderic Acid Biosynthesis in Response to Methyl Jasmonate in Ganoderma Lucidum
Source: Int J Mol Sci. 2019 Dec 4;20(24):6116. doi: 10.3390/ijms20246116 (PMC6941157; doi:10.3390/ijms20246116)
Supplement: Supplementary file 1 [file ijms-20-06116-s001.zip › ijms-642095-revised-r1-supplementary/Table S1-S12/Table S8.docx]

Table S1. The primers used in this study

| Gene name | Forward sequence (5′ to 3′) | Reverse sequence (5′ to 3′) |
| --- | --- | --- |
| *PP2A* | CCTTATGGGCGACAACC | GCATTACCTCGTCAAACAGAT |
| *slt2* | CCACCCTTACCTTGCTGTTT | CTTTCTTGCCGACGGATTT |
| *fus* | GGACCGAGACAGGGTTCAT | TGCAGCCGACCGACCAAAT |
| *pkc* | CGGAGGGACACGGACAT | GGATAGAGCGGCTAAGACAG |
| *ras* | CCCAGTTATCGTCGTTGC | CCAGTCTGCTGCTCCTTG |
| *noxa* | CGTAGAGTTCTTCTGGGTTTGC | CTGCGTGAGGTAGATGTTGATA |
| *hmgr* | GTCATCCTCCTATGCCAAAC | GGGCGTAGTCGTAGTCCTTC |
| *fps* | CCTCATCACCGCTCCAGAA | AGGGCGACGGGAAGGTAGAA |
| *sqs* | AGGGCGACGGGAAGGTAGAA | CGTAGTGGCAGTAGAGGTTG |
| *osc* | AGGGAGAACCCGAAGCATT | CGTCCACAGCGTCGCATAAC |
| Energy metabolism pathway | | |
| HXK2 | CTCACGCTACCAAGAAGACT | GGGATCATAGGCACAACCT |
| PGM2 | AAGGCGGATGGTGGTAT | CGATGACCCTGTATGTGGA |
| HXT10 | CTCTTTCACTCGGGCTTTC | TTGTTTGTGGCGATGTTG |
| TPI1 | CTGACCGAAGCTGACTGG | ACCGCCGTAGATGATGC |
| GCY1 | AGTTACGGTATGTTCAAAGTC | ACGGAAGGGATGTCTGC |
| ENO1 | TTCACGAGGCGGTTGAG | TTGCCCTTGTTCGGAGTA |
| PCK1 | ACAACATGCTTATCCGTCCTA | ACACCCTTCTTCATCTCACC |
| PYC1 | CGCAACAAGCCGAAGAT | CGCTCAAGTCGCCATAC |
| PGK1 | GGTGCCAGCGTAGTCGT | CCAAATCGGATGGTCGT |
| ICL1 | TCGTGAGCGTGGTTTCC | GTGATCCAGACCTGGTTGC |
| Triterpenoid synthesis pathway | | |
| ERG11 | AAGCGAACTCCTCTGCC | GAGCCTCCCTGATCTCC |
| ERG20 | TGTGGTGGCCTGGTTCA | ATGTCGTCGGAAATAAGGAA |
| ERG5 | AGCCGTCGCTGTTTATCG | CCTGCATCGCCTTCGTT |
| ERG13 | CCATCAACGCTTCCACCAG | GCTCCTCCTCCGAAATGC |
| Transcriptional regulation | | |
| TFC6 | CCCCTTGCTCGCTAATCT | CAGGCGTGGCAGTGATGT |
| SIN3 | CCCAATCCGTCGTCAAT | GCTTCAACCCGAACATAAA |
| GIS1 | TTGGTCAACAACGGTAAAG | TCACCCAGTCGAGATAAGG |
| RIM101 | TCCGTCATCAACAGCAACC | GCCTTCAGCCCAAAGAACA |
| Translational regulation | | |
| DBP5 | CCGTCTCCTACATCCACAA | GGACTACGGGCAGACACTT |
| PRP46 | CACCACCCGTCGCTTTA | ACGCACGCATGATACCC |
| RPL6A | AACCTCCGTTCCTCCATC | AGCAACAGTCCGCTCCC |
| RPS6A | CTCATCTCCCGTTGTGCC | GTCGTACTCCGTCTTCTGCT |
| ESS1 | GGTGATTGCGATCTGACTG | CCAAGAAACCGAAATGCTC |
| TIF34 | GGCAAGATTTCGCTATTC | GTGCGTGTCTCGTGGAT |
| Oxidoreduction process | | |
| ADH1 | CGTACATGCGGTCTAAGATG | ACACGGAACTGCGATGC |
| ADH5 | ACGGACAAGAGCAAGAGGG | GCTTCAGGCGGTCCAAAC |
| AGC1 | CCTTCCGTTACCTCATCG | AAATAGAGTACGCAACACCAG |
| CCP1 | CCTCGTCCAGGAGCAGA | ATGAAGGCAGCGAAAGC |
| ATM1 | GGAAGACACCGCTCAACTC | CCGACGATGACGCAAAG |
| CTT1 | TCACGCACGACATCAGC | TCTTGACAGCAAAGCCAC |
| GTT2 | TTAGCACCTGCCACCAT | TGTCGCTGTCGGATAGAA |
| FMS1 | GGGTGAAGGGTTCGTGG | ATCTTCGGGTCGCCAAT |
