# Supplementary figures and images for: Integrated Proteomics and Metabolomics Analysis Provides Insights into Ganoderic Acid Biosynthesis in Response to Methyl Jasmonate in Ganoderma Lucidum
Source: Int J Mol Sci. 2019 Dec 4;20(24):6116. doi: 10.3390/ijms20246116 (PMC6941157; doi:10.3390/ijms20246116)

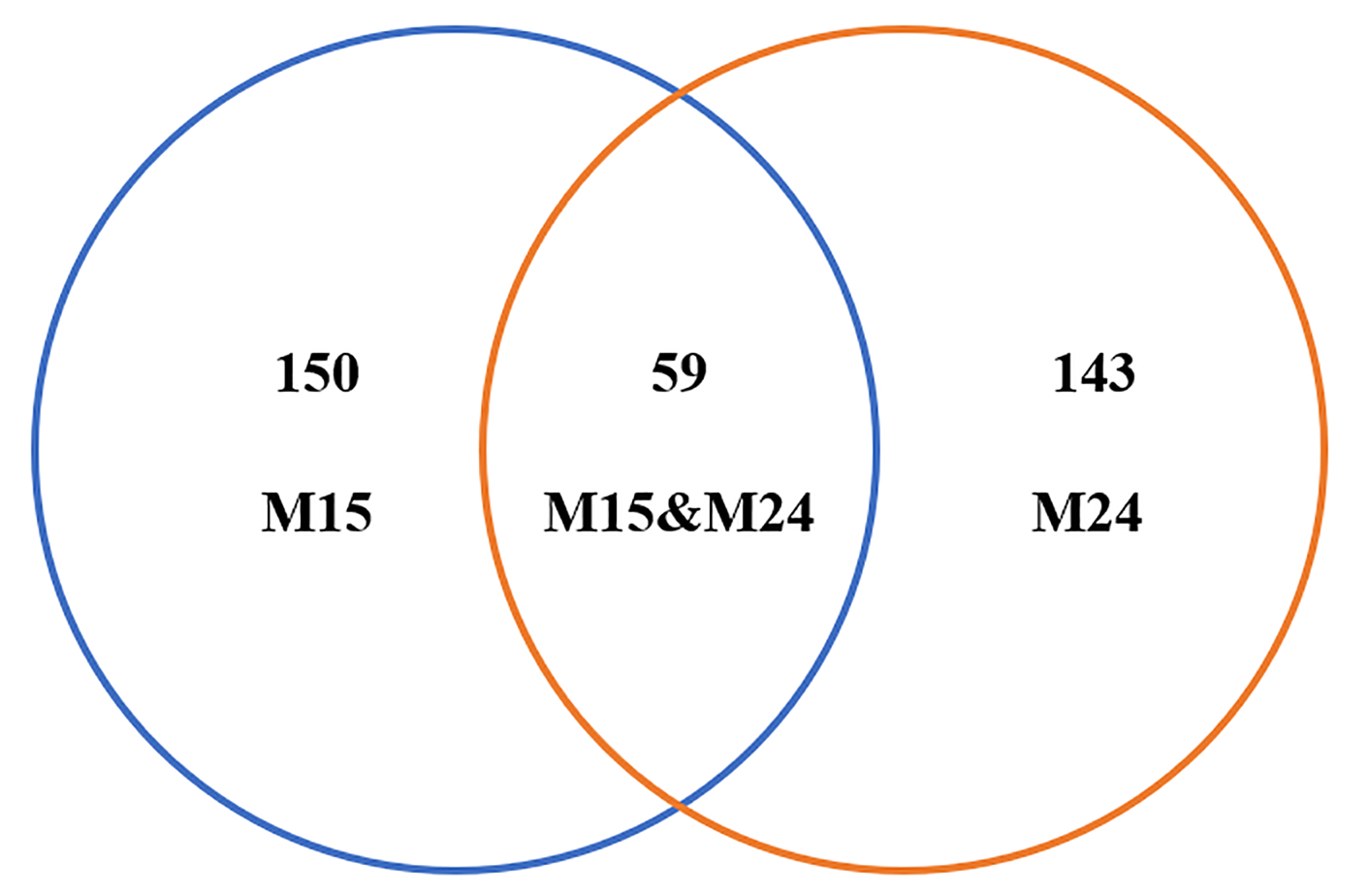

Supplement: Supplementary file 1 [file ijms-20-06116-s001.zip › ijms-642095-revised-r1-supplementary/Figure S1-S7/Fig. S1 Venn.tif]

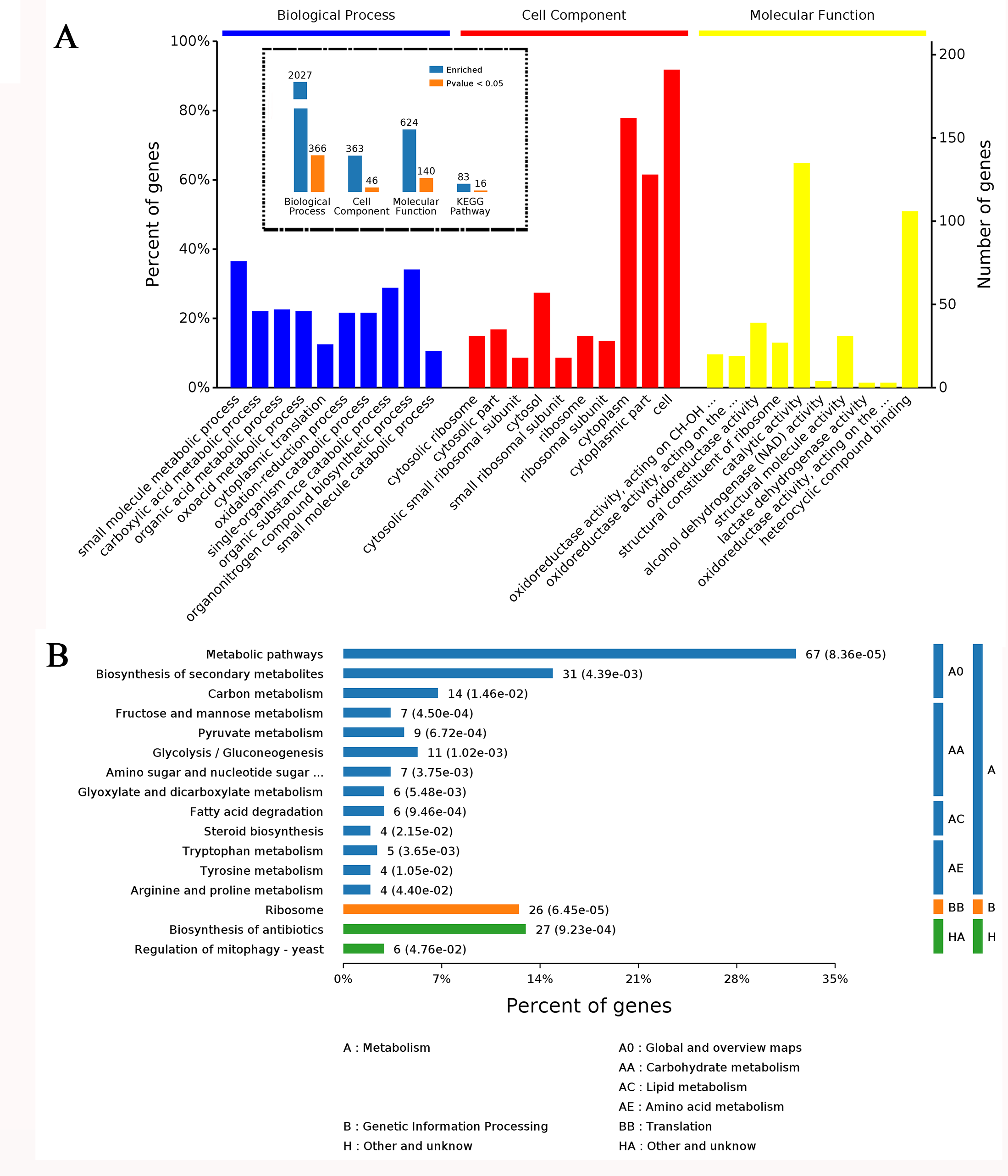

Supplement: Supplementary file 1 [file ijms-20-06116-s001.zip › ijms-642095-revised-r1-supplementary/Figure S1-S7/Fig. S2 M15 GO and KEGG.tif]

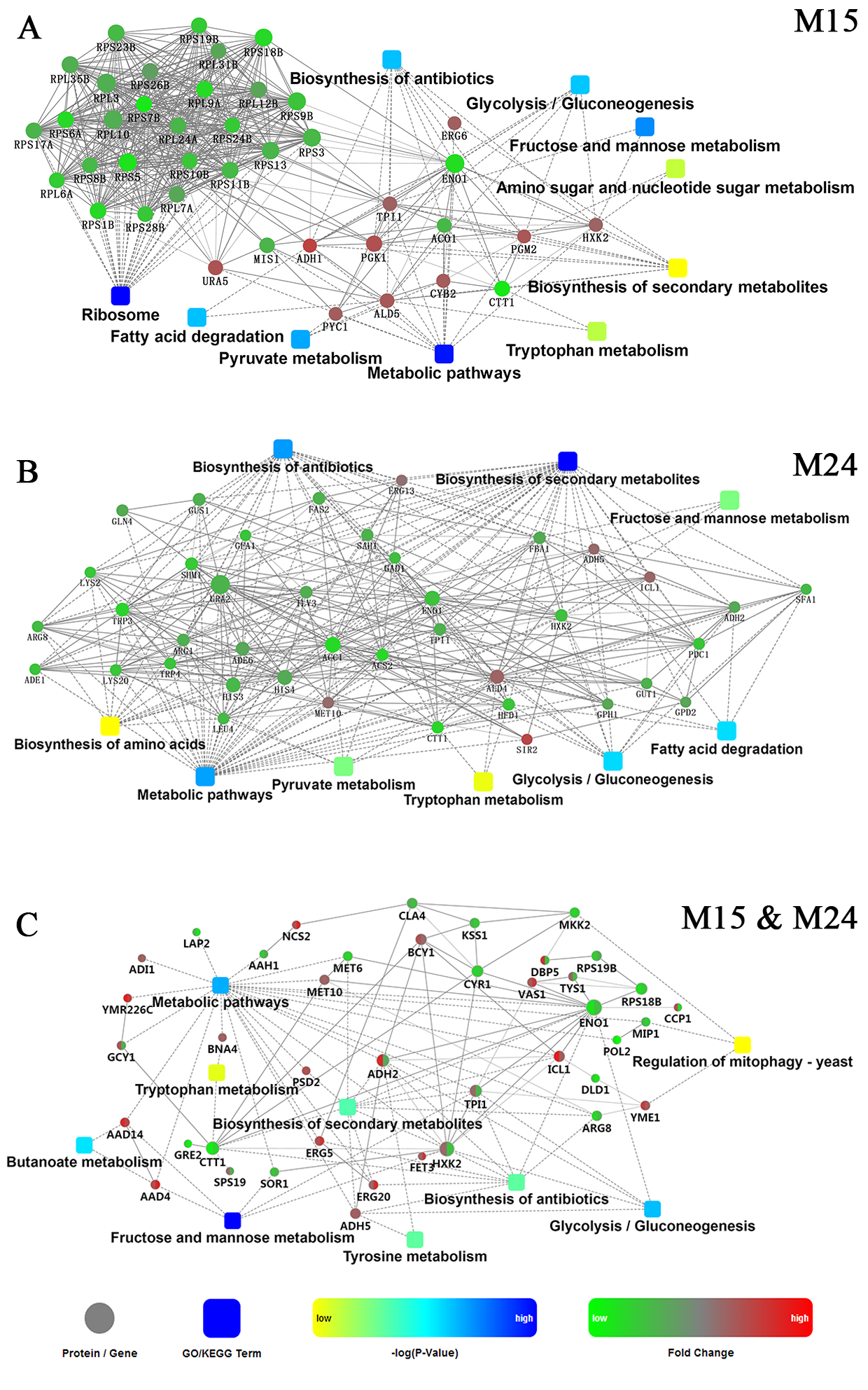

Supplement: Supplementary file 1 [file ijms-20-06116-s001.zip › ijms-642095-revised-r1-supplementary/Figure S1-S7/Fig. S3 Bioinformatics analysis .tif]

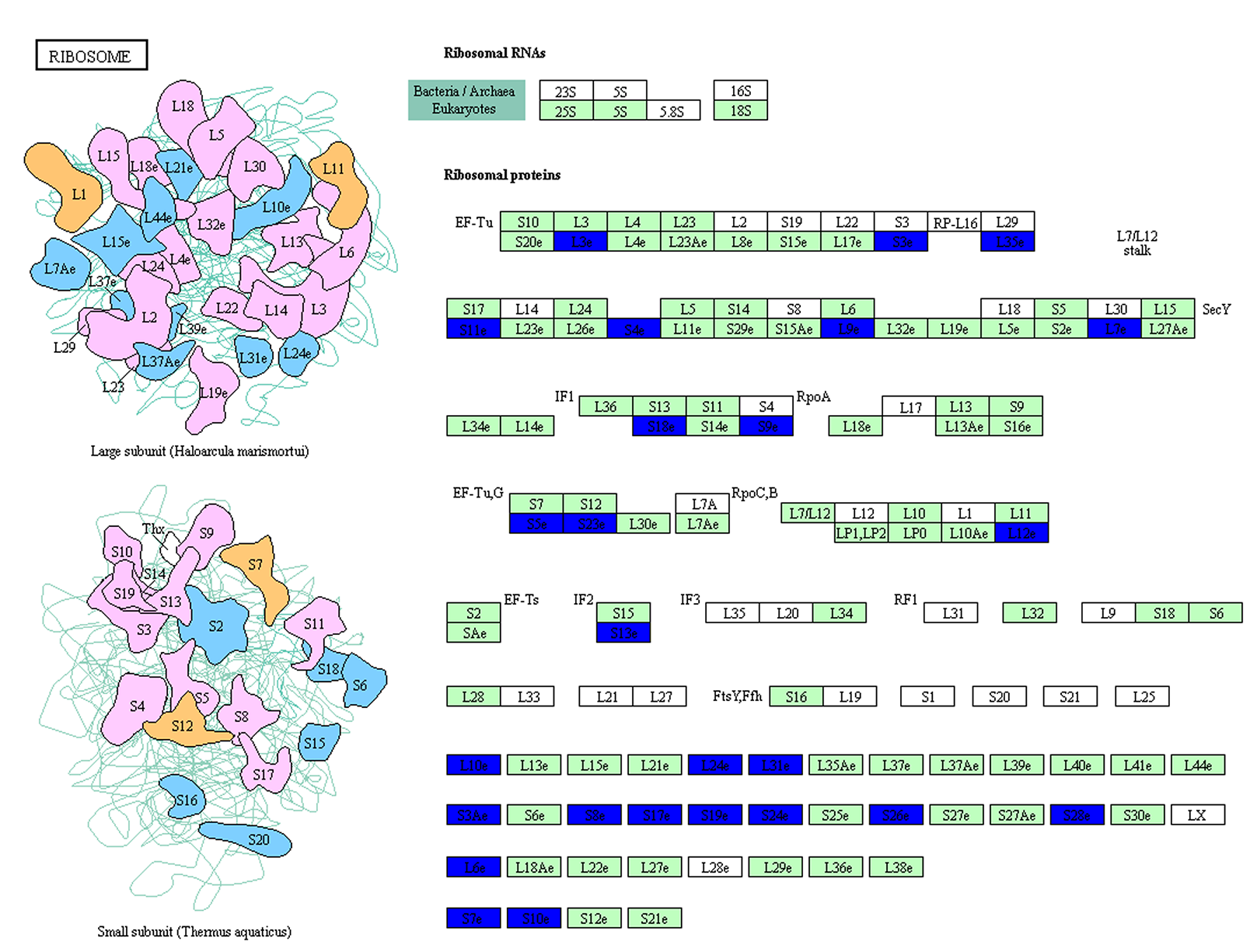

Supplement: Supplementary file 1 [file ijms-20-06116-s001.zip › ijms-642095-revised-r1-supplementary/Figure S1-S7/Fig. S4 ribosome .tif]

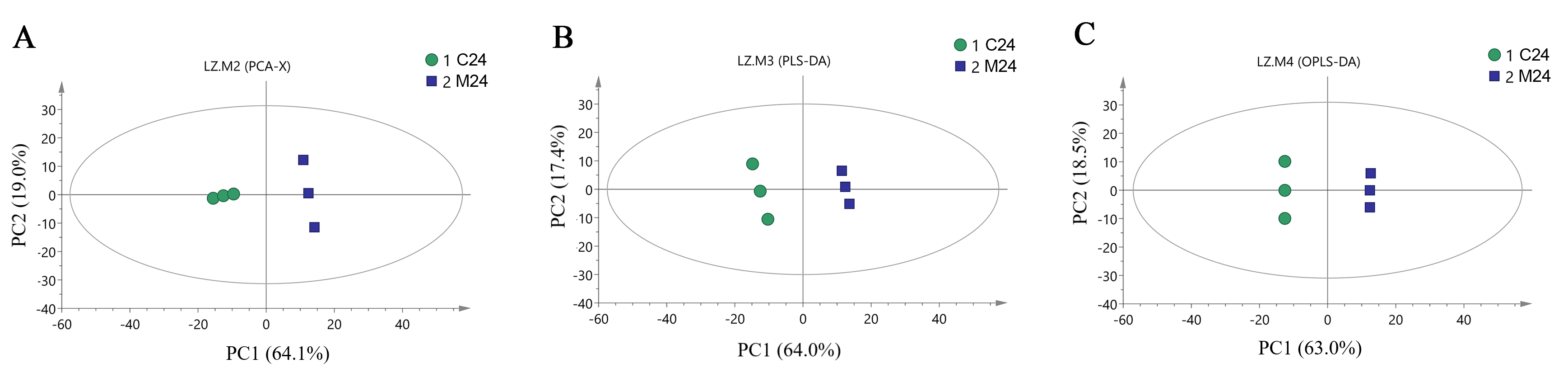

Supplement: Supplementary file 1 [file ijms-20-06116-s001.zip › ijms-642095-revised-r1-supplementary/Figure S1-S7/Fig. S5 GC PCA PLS .tif]

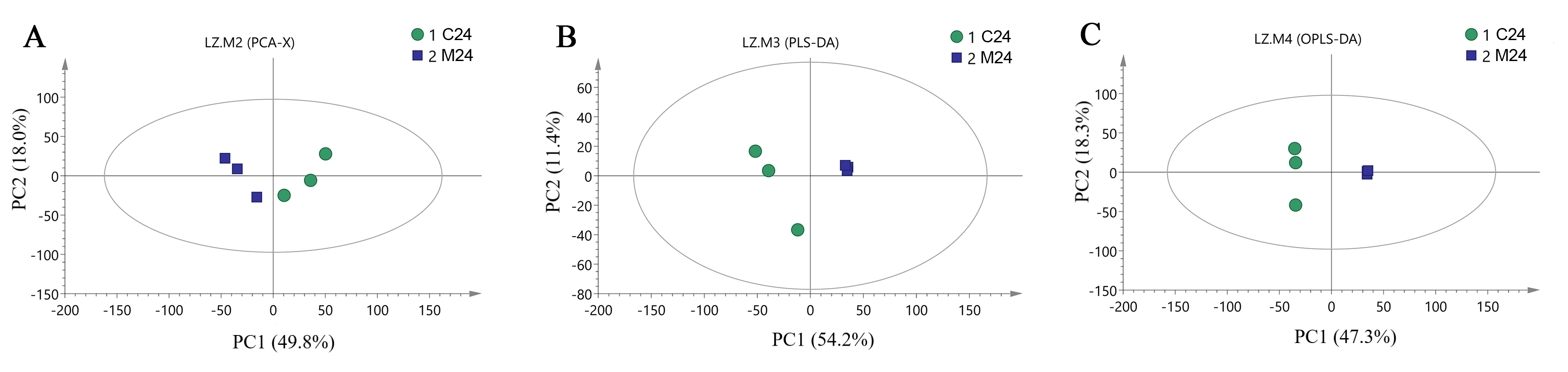

Supplement: Supplementary file 1 [file ijms-20-06116-s001.zip › ijms-642095-revised-r1-supplementary/Figure S1-S7/Fig. S6 LC PCA PLS .tif]

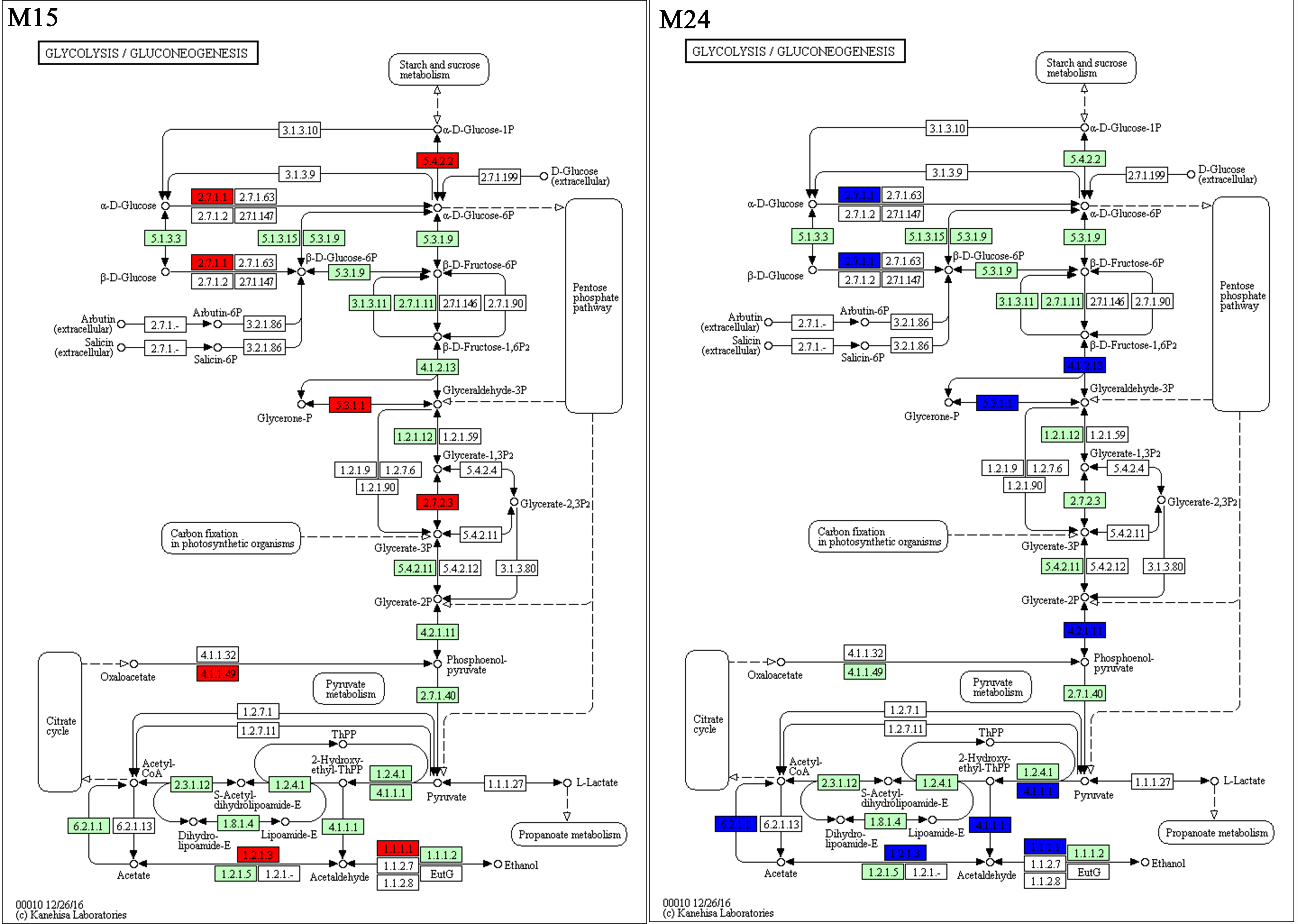

Supplement: Supplementary file 1 [file ijms-20-06116-s001.zip › ijms-642095-revised-r1-supplementary/Figure S1-S7/Fig. S7 EMP of M15 and M24.tif]
