# Supplementary material for: Integrated Proteomics and Metabolomics Analysis Provides Insights into Ganoderic Acid Biosynthesis in Response to Methyl Jasmonate in Ganoderma Lucidum
Source: Int J Mol Sci. 2019 Dec 4;20(24):6116. doi: 10.3390/ijms20246116 (PMC6941157; doi:10.3390/ijms20246116)
Supplement: Supplementary file 1 [file ijms-20-06116-s001.zip › ijms-642095-revised-r1-supplementary/Table S1-S12/Table S2 up M15.doc]

Table S2 Up-regulated differential expressed proteins of MeJA treatment for 15 min

| **NO.** | **GL-ID** | **Fold Change** | **Uniprot ID** | **Gene name** | **Protein description** |
| --- | --- | --- | --- | --- | --- |
| 1 | GL22360-R1_1 | 5.31 | Q07993 | XYL2 | D-xylulose reductase |
| 2 | GL22268-R1_1 | 1.762 | P00942 | TPI1 | Triosephosphate isomerase |
| 3 | GL20491-R1_1 | 1.683 | P04807 | HXK2 | Hexokinase-2 |
| 4 | GL24280-R1_1 | 2.136 | P37012 | PGM2 | Phosphoglucomutase 2 |
| 5 | GL27042-R1_1 | 3.233 | P54781 | ERG5 | Cytochrome P450 61 |
| 6 | GL16838-R1_1 | 2.006 | P53199 | ERG26 | Sterol-4-alpha-carboxylate 3-dehydrogenase, decarboxylating |
| 7 | GL28943-R1_1 | 5.514 | P10614 | ERG11 | Lanosterol 14-alpha demethylase |
| 8 | GL28304-R1_1 | 1.808 | P25087 | ERG6 | Sterol 24-C-methyltransferase |
| 9 | GL29918-R1_1 | 2.048 | P40009 | YND1 | Golgi apyrase |
| 10 | GL16081-R1_1 | 2.354 | Q03677 | ADI1 | 1,2-dihydroxy-3-keto-5-methylthiopentene dioxygenase |
| 11 | GL26572-R1_1 | 2.065 | P40047 | ALD5 | Aldehyde dehydrogenase 5, mitochondrial |
| 12 | GL24618-R1_1 | 2.586 | P47039 | BNA3 | Probable kynurenine--oxoglutarate transaminase BNA3 |
| 13 | GL27379-R1_1 | 2.131 | P38169 | BNA4 | Kynurenine 3-monooxygenase |
| 14 | GL28558-R1_1 | 1.697 | Q05979 | BNA5 | Kynureninase |
| 15 | GL19331-R1_1 | 1.919 | P32603 | SPR1 | Sporulation-specific glucan 1,3-beta-glucosidase |
| 16 | GL23580-R1_1 | 2.171 | P08019 | SGA1 | Glucoamylase, intracellular sporulation-specific |
| 17 | GL22281-R1_1 | 2.04 | P38626 | CBR1 | NADH-cytochrome b5 reductase 1 |
| 18 | GL22188-R1_1 | 1.779 | Q06350 | CTS2 | Sporulation-specific chitinase 2 |
| 19 | GL30088-R1_1 | 2.51 | P43123 | QRI1 | UDP-N-acetylglucosamine pyrophosphorylase |
| 20 | GL22224-R1_1 | 2.528 | Q08108 | PLB3 | Lysophospholipase 3 |
| 21 | GL18800-R1_1 | 3.3 | P40471 | AYR1 | NADPH-dependent 1-acyldihydroxyacetone phosphate reductase |
| 22 | GL15153-R1_1 | 2.932 | P53037 | PSD2 | Phosphatidylserine decarboxylase proenzyme 2 |
| 23 | GL24805-R1_1 | 3.69 | P46681 | DLD2 | D-2-hydroxyglutarate--pyruvate transhydrogenase DLD2 |
| 24 | GL21586-R1_1 | 1.941 | P00175 | CYB2 | Cytochrome b2, mitochondrial |
| 25 | GL22784-R1_1 | 7.68 | Q08911 | FDH1 | Formate dehydrogenase 1 |
| 26 | GL28863-R1_1 | 7.869 | P42884 | AAD14 | Putative aryl-alcohol dehydrogenase AAD14 |
| 27 | GL24071-R2_1 | 3.377 | Q07747 | AAD4 | Probable aryl-alcohol dehydrogenase AAD4 |
| 28 | GL26703-R1_1 | 4.602 | Q03778 | FMN1 | Riboflavin kinase |
| 29 | GL25862-R1_1 | 1.894 | P39683 | NPT1 | Nicotinate phosphoribosyltransferase |
| 30 | GL21958-R1_1 | 1.772 | P38795 | QNS1 | Glutamine-dependent NAD(+) synthetase |
| 31 | GL22756-R1_1 | 1.656 | P21373 | UTR1 | NAD(+) kinase |
| 32 | GL18370-R1_1 | 2.227 | P47051 | AIM22 | Putative lipoate-protein ligase A |
| 33 | GL17808-R1_1 | 3.306 | P24521 | ERG8 | Phosphomevalonate kinase |
| 34 | GL22068-R1_1 | 1.787 | P08524 | ERG20 | Farnesyl pyrophosphate synthase |
| 35 | GL20687-R1_1 | 1.999 | P39692 | MET10 | Sulfite reductase [NADPH] flavoprotein component |
| 36 | GL15300-R1_1 | 3.818 | P48526 | ISM1 | Isoleucine--tRNA ligase, mitochondrial |
| 37 | GL26118-R1_1 | 2.754 | P40825 | ALA1 | Alanine--tRNA ligase, mitochondrial |
| 38 | GL23738-R1_1 | 2.43 | P07806 | VAS1 | Valine--tRNA ligase, mitochondrial |
| 39 | GL27820-R1_1 | 1.973 | P36421 | TYS1 | Tyrosine--tRNA ligase, cytoplasmic |
| 40 | GL24308-R1_1 | 8.334 | Q06549 | CDD1 | Cytidine deaminase |
| 41 | GL24054-R1_1 | 2.268 | P13298 | URA5 | Orotate phosphoribosyltransferase 1 |
| 42 | GL18836-R1_1 | 1.832 | P07258 | CPA1 | Carbamoyl-phosphate synthase arginine-specific small chain |
| 43 | GL16823-R1_1 | 9.697 | Q05016 | YMR226C | NADP-dependent 3-hydroxy acid dehydrogenase |
| 44 | GL24473-R1_1 | 2.719 | P14065 | GCY1 | Glycerol 2-dehydrogenase |
| 45 | GL25184-R1_1 | 2.587 | P25641 | ATG15 | Putative lipase ATG15 |
| 46 | GL25157-R2_1 | 1.829 | Q05567 | DPL1 | Sphingosine-1-phosphate lyase |
| 47 | GL31396-R1_1 | 4.555 | P38787 | PAN5 | 2-dehydropantoate 2-reductase |
| 48 | GL25620-R1_1 | 5.103 | P10963 | PCK1 | Phosphoenolpyruvate carboxy kinase (ATP) |
| 49 | GL18758-R1_1 | 1.635 | P43567 | AGX1 | Alanine--glyoxylate aminotransferase 1 |
| 50 | GL24613-R1_1 | 1.92 | P00812 | CAR1 | Arginase |
| 51 | GL20764-R1_1 | 7.821 | P00331 | ADH2 | Alcohol dehydrogenase 2 |
| 52 | GL22020-R1_1 | 4.721 | P07246 | ADH3 | Alcohol dehydrogenase 3, mitochondria |
| 53 | GL24208-R1_1 | 3.082 | P00330 | ADH1 | Alcohol dehydrogenase 1 |
| 54 | GL24163-R1_1 | 2.188 | P38113 | ADH5 | Alcohol dehydrogenase 5 |
| 55 | GL27739-R1_1 | 2.38 | P00560 | PGK1 | Phosphoglycerate kinase 1 |
| 56 | GL22748-R1_1 | 2.086 | Q05911 | ADE13 | Adenylosuccinate lyase |
| 57 | GL16921-R1_1 | 2.426 | P32178 | ARO7 | Chorismate mutase |
| 58 | GL22666-R1_1 | 2.504 | P36013 | MAE1 | NAD-dependent malic enzyme, mitochondrial |
| 59 | GL23465-R1_1 | 3.79 | P21826 | DAL7 | Malate synthase 2, glyoxysomal |
| 60 | GL21539-R1_1 | 10.608 | P28240 | ICL1 | Isocitrate lyase |
| 61 | GL21264-R1_1 | 2.187 | P39525 | CEM1 | 3-oxoacyl-[acyl-carrier-protein] synthase homolog |
| 62 | GL21673-R1_1 | 1.874 | P11154 | PYC1 | Pyruvate carboxylase 1 |
| 63 | GL23892-R1_1 | 1.948 | P12866 | STE6 | Alpha-factor-transporting ATPase |
| 64 | GL23083-R1_1 | 2.307 | Q12019 | MDN1 | Midasin |
| 65 | GL29522-R1_1 | 1.914 | P32794 | AFG2 | ATPase family gene 2 protein |
| 66 | GL17345-R1_1 | 2.94 | P38792 | RRP4 | Exosome complex component RRP4 |
| 67 | GL26068-R1_1 | 2.162 | P35207 | SKI2 | Antiviral helicase SKI2 |
| 68 | GL26021-R1_1 | 4.563 | P25336 | MSH3 | DNA mismatch repair protein MSH3 |
| 69 | GL25600-R1_1 | 2.025 | P12630 | BAR1 | Barrierpepsin |
| 70 | GL27858-R1_1 | 2.124 | Q12215 | WSC3 | Cell wall integrity and stress response component 3 |
| 71 | GL23980-R1_1 | 1.808 | P06787 | CMD1 | Calmodulin |
| 72 | GL22664-R1_1 | 5.488 | P25389 | KCC4 | Probable serine/threonine-protein kinase KCC4 |
| 73 | GL16966-R1_1 | 2.145 | P54790 | ORC3 | Origin recognition complex subunit 3 |
| 74 | GL22379-R1_1 | 1.791 | Q12440 | APC2 | Anaphase-promoting complex subunit 2 |
| 75 | GL21226-R1_1 | 4.588 | P38013 | AHP1 | Peroxiredoxin AHP1 |
| 76 | GL25874-R1_1 | 3.345 | P53923 | NCS2 | Cytoplasmic tRNA 2-thiolation protein 2 |
| 77 | GL25929-R1_1 | 1.875 | P32912 | VAM7 | Vacuolar morphogenesis protein 7 |
| 78 | GL17930-R1_1 | 4.786 | P32795 | YME1 | Mitochondrial inner membrane i-AAA protease supercomplex subunit YME1 |
| 79 | GL23165-R1_1 | 1.667 | P32361 | IRE1 | Serine/threonine-protein kinase/endoribonuclease IRE1 |
| 80 | GL21371-R1_1 | 1.776 | P16474 | KAR2 | 78 kDa glucose-regulated protein homolog |
| 81 | GL31667-R1_1 | 6.562 | Q00381 | APS2 | AP-2 complex subunit sigm |
| 82 | GL22662-R1_1 | 1.684 | P32573 | SPS19 | Peroxisomal 2,4-dienoyl-CoA reductase SPS19 |
| 83 | GL23589-R1_1 | 1.82 | P39518 | FAA2 | Long-chain-fatty-acid--CoA ligase 2 |
| 84 | GL25668-R1_1 | 2.214 | Q03833 | GIS1 | Transcriptional activator/repressor GIS1 |
| 85 | GL23646-R1_1 | 1.917 | Q12482 | AGC1 | Mitochondrial aspartate-glutamate transporter AGC1 |
| 86 | GL22266-R1_1 | 4.548 | Q12340 | YRM1 | Zinc finger transcription factor YRM1 |
| 87 | GL23368-R1_1 | 2.116 | Q08300 | YOL159C | Uncharacterized protein YOL159C |
| 88 | GL17746-R1_1 | 6.17 | Q06339 | TFC6 | Transcription factor tau 91 kDa subunit |
| 89 | GL21210-R1_1 | 2.528 | Q06010 | STE23 | A-factor-processing enzyme |
| 90 | GL20443-R1_1 | 1.856 | Q04585 | YDR109C | Uncharacterized sugar kinase YDR109C |
| 91 | GL23275-R1_1 | 2.527 | Q04279 | SEG1 | Eisosome protein SEG1 |
| 92 | GL24915-R1_1 | 1.939 | Q03656 | SKY1 | Serine/threonine-protein kinase SKY1 |
| 93 | GL30597-R1_1 | 2.187 | Q02933 | RNY1 | Ribonuclease T2-like |
| 94 | GL28773-R1_1 | 3.577 | Q02875 | SYH1 | SMY2 homolog 2 |
| 95 | GL30277-R1_1 | 1.683 | Q00245 | RHO3 | GTP-binding protein RHO3 |
| 96 | GL21563-R1_1 | 2.042 | P53839 | GOR1 | Glyoxylate reductase 1 |
| 97 | GL31536-R1_1 | 2.679 | P53819 | YRF1-6 | Y' element ATP-dependent helicase protein 1 copy 6 |
| 98 | GL23079-R1_1 | 2.476 | P53195 | COG7 | Conserved oligomeric Golgi complex subunit 7 |
| 99 | GL30780-R1_1 | 1.769 | P47172 | YJR141W | Uncharacterized protein YJR141W |
| 100 | GL23379-R1_1 | 10.201 | P46683 | YAR1 | Ankyrin repeat-containing protein YAR1 |
| 101 | GL25647-R1_1 | 2.062 | P39010 | AKR1 | Palmitoyltransferase AKR1 |
| 102 | GL16398-R1_1 | 1.901 | P38993 | FET3 | Iron transport multicopper oxidase FET3 |
| 103 | GL24373-R1_1 | 2.628 | P38814 | SBE22 | Protein SBE22 |
| 104 | GL21373-R1_1 | 1.902 | P38260 | FES1 | Hsp70 nucleotide exchange factor FES1 |
| 105 | GL21296-R1_1 | 1.768 | P38254 | PBY1 | Probable tubulin--tyrosine ligase PBY1 |
| 106 | GL21688-R1_1 | 8.309 | P38198 | STU1 | Protein STU1 |
| 107 | GL21285-R2_1 | 1.931 | P37302 | APE3 | Aminopeptidase Y |
| 108 | GL30269-R1_1 | 5.648 | P32857 | PTM1 | Membrane protein PTM1 |
| 109 | GL16905-R1_1 | 1.995 | P32579 | SUA5 | Threonylcarbamoyl-AMP synthase |
| 110 | GL20904-R1_1 | 2.262 | P32499 | NUP2 | Nucleoporin NUP2 |
| 111 | GL19968-R1_1 | 2.686 | P22696 | ESS1 | Peptidyl-prolyl cis-trans isomerase ESS1 |
| 112 | GL29873-R1_1 | 2.502 | P22146 | GAS1 | 1,3-beta-glucanosyltransferase GAS1 |
| 113 | GL26639-R1_1 | 6.018 | P20449 | DBP5 | ATP-dependent RNA helicase DBP5 |
| 114 | GL20763-R1_1 | 1.866 | P15891 | ABP1 | Actin-binding protein |
| 115 | GL30364-R1_1 | 1.852 | P08638 | LEU3 | Regulatory protein LEU3 |
| 116 | GL20414-R1_1 | 3 | P07278 | BCY1 | cAMP-dependent protein kinase regulatory subunit |
| 117 | GL25664-R1_1 | 2.786 | P07267 | PEP4 | Saccharopepsin |
| 118 | GL23456-R1_1 | 2.856 | P00431 | CCP1 | Cytochrome c peroxidase, mitochondrial |
