# Supplementary material for: Integrated Proteomics and Metabolomics Analysis Provides Insights into Ganoderic Acid Biosynthesis in Response to Methyl Jasmonate in Ganoderma Lucidum
Source: Int J Mol Sci. 2019 Dec 4;20(24):6116. doi: 10.3390/ijms20246116 (PMC6941157; doi:10.3390/ijms20246116)
Supplement: Supplementary file 1 [file ijms-20-06116-s001.zip › ijms-642095-revised-r1-supplementary/Table S1-S12/Table S3 down M15.doc]

Table S3 Down-regulated differential expressed proteins of MeJA treatment for 15min

| **NO.** | **GL-ID** | **Fold Change** | **Uniprot ID** | **Genename** | **Protein description** |
| --- | --- | --- | --- | --- | --- |
| 1 | GL28711-R1_1 | 0.397 | P11655 | SEC12 | Guanine nucleotide-exchange factor SEC12 |
| 2 | GL24313-R1_1 | 0.451 | P40083 | YER137C | Uncharacterized protein YEL137C |
| 3 | GL21407-R1_1 | 0.38 | P43593 | UBP6 | Ubiquitin carboxyl-terminal hydrolase 6 |
| 4 | GL22129-R1_1 | 0.486 | P31115 | DEG1 | tRNA pseudouridine(38/39) synthase |
| 5 | GL24127-R1_1 | 0.524 | P22579 | SIN3 | Transcriptional regulatory protein SIN3 |
| 6 | GL16218-R1_1 | 0.503 | P47079 | CCT8 | T-complex protein 1 subunit theta |
| 7 | GL25217-R1_1 | 0.248 | P40061 | TSC11 | Target of rapamycin complex 2 subunit TSC11 |
| 8 | GL21054-R1_1 | 0.43 | P17883 | SKI3 | Superkiller protein 3 |
| 9 | GL19851-R1_1 | 0.6 | Q12267 | SMC4 | Structural maintenance of chromosomes protein 4 |
| 10 | GL31661-R1_1 | 0.405 | P35497 | SOR1 | Sorbitol dehydrogenase 1 |
| 11 | GL30701-R1_1 | 0.643 | Q12118 | SGT2 | Small glutamine-rich tetratricopeptide repeat-containing protein 2 |
| 12 | GL23680-R1_1 | 0.297 | P0CY13 | HMRA2 | Silenced mating-type protein A2 |
| 13 | GL23736-R1_1 | 0.295 | Q08299 | ENB1 | Siderophore iron transporter ENB1 |
| 14 | GL27917-R1_1 | 0.528 | P48562 | CLA4 | Serine/threonine-protein kinase CLA4 |
| 15 | GL22546-R1_1 | 0.578 | Q12159 | YRA1 | RNA annealing protein YRA1 |
| 16 | GL20422-R1_1 | 0.525 | P38630 | RFC1 | Replication factor C subunit 1 |
| 17 | GL23734-R1_1 | 0.535 | P24583 | PKC1 | Protein kinase C-like 1 |
| 18 | GL30840-R1_1 | 0.496 | P43535 | GCN20 | Protein GCN20 |
| 19 | GL25322-R1_1 | 0.489 | Q12417 | PRP46 | Pre-mRNA-splicing factor PRP46 |
| 20 | GL30526-R1_1 | 0.324 | P50264 | FMS1 | Polyamine oxidase FMS1 |
| 21 | GL26114-R1_1 | 0.307 | P53297 | PBP1 | PAB1-binding protein 1 |
| 22 | GL24116-R1_1 | 0.477 | P32833 | ORC2 | Origin recognition complex subunit 2 |
| 23 | GL17170-R1_1 | 0.337 | P33327 | GDH2 | NAD-specific glutamate dehydrogenase |
| 24 | GL16520-R1_1 | 0.311 | Q12068 | GRE2 | NADPH-dependent methylglyoxal reductase GRE2 |
| 25 | GL18913-R1_1 | 0.408 | P36060 | MCR1 | NADH-cytochrome b5 reductase 2 |
| 26 | GL24090-R1_1 | 0.628 | Q03162 | MUB1 | MYND-type zinc finger protein MUB1 |
| 27 | GL19525-R1_1 | 0.383 | Q9P305 | IGO2 | mRNA stability protein IGO2 |
| 28 | GL24589-R1_1 | 0.473 | P14681 | KSS1 | Mitogen-activated protein kinase KSS1 |
| 29 | GL22372-R1_1 | 0.562 | P32332 | OAC1 | Mitochondrial oxaloacetate transport protein |
| 30 | GL20605-R1_1 | 0.316 | P32491 | MKK2 | MAP kinase kinase MKK2/SSP33 |
| 31 | GL22193-R1_1 | 0.621 | P29952 | PMI40 | Mannose-6-phosphate isomerase |
| 32 | GL30169-R1_1 | 0.477 | Q10740 | LAP2 | Leukotriene A-4 hydrolase homolog |
| 33 | GL25521-R1_1 | 0.493 | P24309 | DBR1 | Lariat debranching enzyme |
| 34 | GL22429-R2_1 | 0.57 | P53901 | INN1 | Ingression protein 1 |
| 35 | GL26074-R1_1 | 0.416 | P33307 | CSE1 | Importin alpha re-exporter |
| 36 | GL20749-R1_1 | 0.507 | P43581 | HXT10 | Hexose transporter HXT10 |
| 37 | GL16402-R1_1 | 0.444 | P10961 | HSF1 | Heat shock factor protein |
| 38 | GL16910-R1_1 | 0.573 | P33322 | CBF5 | H/ACA ribonucleoprotein complex subunit 4 |
| 39 | GL30564-R1_1 | 0.4 | Q12390 | GTT2 | Glutathione S-transferase 2 |
| 40 | GL21848-R1_1 | 0.447 | P54885 | PRO2 | Gamma-glutamyl phosphate reductase |
| 41 | GL30114-R1_1 | 0.258 | P00924 | ENO1 | Enolase 1 |
| 42 | GL24443-R1_1 | 0.441 | P33892 | GCN1 | eIF-2-alpha kinase activator GCN1 |
| 43 | GL23367-R1_1 | 0.444 | P15801 | MIP1 | DNA polymerase gamma |
| 44 | GL31410-R1_1 | 0.244 | P21951 | POL2 | DNA polymerase epsilon catalytic subunit A |
| 45 | GL26490-R1_1 | 0.358 | P32891 | DLD1 | D-lactate dehydrogenase [cytochrome] 1, mitochondrial |
| 46 | GL15754-R1_1 | 0.382 | P43550 | DAK2 | Dihydroxyacetone kinase 2 |
| 47 | GL28021-R1_1 | 0.557 | P38344 | REI1 | Cytoplasmic 60S subunit biogenesis factor REI1 |
| 48 | GL24643-R1_1 | 0.265 | Q02554 | CUS1 | Cold sensitive U2 snRNA suppressor 1 |
| 49 | GL29415-R1_1 | 0.394 | Q03124 | RSC9 | Chromatin structure-remodeling complex subunit RSC9 |
| 50 | GL17946-R1_1 | 0.594 | P41695 | BUB1 | Checkpoint serine/threonine-protein kinase BUB1 |
| 51 | GL30629-R1_1 | 0.372 | P32457 | CDC3 | Cell division control protein 3 |
| 52 | GL24518-R1_1 | 0.518 | P25342 | CDC10 | Cell division control protein 10 |
| 53 | GL22189-R1_1 | 0.223 | P06115 | CTT1 | Catalase T |
| 54 | GL24693-R1_1 | 0.469 | P09440 | MIS1 | C-1-tetrahydrofolate synthase, mitochondrial |
| 55 | GL23021-R1_1 | 0.514 | P38009 | ADE17 | Bifunctional purine biosynthesis protein ADE17 |
| 56 | GL26084-R1_1 | 0.157 | P46682 | APL6 | AP-3 complex subunit beta |
| 57 | GL30014-R1_1 | 0.422 | P53886 | APC1 | Anaphase-promoting complex subunit 1 |
| 58 | GL17791-R1_1 | 0.487 | Q07959 | IZH3 | ADIPOR-like receptor IZH3 |
| 59 | GL28407-R1_1 | 0.328 | P08678 | CYR1 | Adenylate cyclase |
| 60 | GL24512-R1_1 | 0.368 | P53909 | AAH1 | Adenine deaminase |
| 61 | GL18572-R1_1 | 0.448 | P19414 | ACO1 | Aconitate hydratase, mitochondrial |
| 62 | GL19179-R1_1 | 0.291 | P18544 | ARG8 | Acetylornithine aminotransferase, mitochondrial |
| 63 | GL31662-R1_1 | 0.331 | P05694 | MET6 | 5-methyltetrahydropteroyltriglutamate--homocysteine methyltransferase |
| 64 | GL31416-R1_1 | 0.271 | P05738 | RPL9A | 60S ribosomal protein L9-A |
| 65 | GL24546-R1_1 | 0.571 | P05737 | RPL7A | 60S ribosomal protein L7-A |
| 66 | GL31415-R1_1 | 0.334 | Q02326 | RPL6A | 60S ribosomal protein L6-A |
| 67 | GL31430-R1_1 | 0.505 | P0CX85 | RPL35B | 60S ribosomal protein L35-B |
| 68 | GL20960-R1_1 | 0.571 | P0C2H9 | RPL31B | 60S ribosomal protein L31-B |
| 69 | GL31324-R1_1 | 0.511 | P14126 | RPL3 | 60S ribosomal protein L3 |
| 70 | GL22280-R1_1 | 0.461 | P04449 | RPL24A | 60S ribosomal protein L24-A |
| 71 | GL20175-R1_1 | 0.579 | P0CX54 | RPL12B | 60S ribosomal protein L12-B |
| 72 | GL25139-R1_1 | 0.515 | P41805 | RPL10 | 60S ribosomal protein L10 |
| 73 | GL30112-R1_1 | 0.376 | P05755 | RPS9B | 40S ribosomal protein S9-B |
| 74 | GL25633-R1_1 | 0.442 | P0CX40 | RPS8B | 40S ribosomal protein S8-B |
| 75 | GL31438-R1_1 | 0.221 | P48164 | RPS7B | 40S ribosomal protein S7-B |
| 76 | GL22318-R2_1 | 0.274 | P0CX37 | RPS6A | 40S ribosomal protein S6-A |
| 77 | GL25761-R1_1 | 0.239 | P26783 | RPS5 | 40S ribosomal protein S5 |
| 78 | GL23016-R1_1 | 0.356 | P0CX36 | RPS4B | 40S ribosomal protein S4-B |
| 79 | GL30248-R1_1 | 0.444 | P05750 | RPS3 | 40S ribosomal protein S3 |
| 80 | GL29915-R1_1 | 0.354 | P0C0X0 | RPS28B | 40S ribosomal protein S28-B |
| 81 | GL24058-R1_1 | 0.642 | P39939 | RPS26B | 40S ribosomal protein S26-B |
| 82 | GL30250-R1_1 | 0.343 | P0CX32 | RPS24B | 40S ribosomal protein S24-B |
| 83 | GL25300-R1_1 | 0.436 | P0CX30 | RPS23B | 40S ribosomal protein S23-B |
| 84 | GL26664-R1_1 | 0.278 | P23248 | RPS1B | 40S ribosomal protein S1-B |
| 85 | GL28146-R1_1 | 0.345 | P07281 | RPS19B | 40S ribosomal protein S19-B |
| 86 | GL25744-R1_1 | 0.277 | P0CX56 | RPS18B | 40S ribosomal protein S18-B |
| 87 | GL30395-R1_1 | 0.472 | P02407 | RPS17A | 40S ribosomal protein S17-A |
| 88 | GL22454-R1_1 | 0.46 | P05756 | RPS13 | 40S ribosomal protein S13 |
| 89 | GL30557-R1_1 | 0.435 | P0CX48 | RPS11B | 40S ribosomal protein S11-B |
| 90 | GL24161-R1_1 | 0.364 | P46784 | RPS10B | 40S ribosomal protein S10-B |
| 91 | GL25982-R1_1 | 0.467 | P40327 | RPT2 | 26S proteasome regulatory subunit 4 |
