# Supplementary material for: Integrated Proteomics and Metabolomics Analysis Provides Insights into Ganoderic Acid Biosynthesis in Response to Methyl Jasmonate in Ganoderma Lucidum
Source: Int J Mol Sci. 2019 Dec 4;20(24):6116. doi: 10.3390/ijms20246116 (PMC6941157; doi:10.3390/ijms20246116)
Supplement: Supplementary file 1 [file ijms-20-06116-s001.zip › ijms-642095-revised-r1-supplementary/Table S1-S12/Table S4 up M24.doc]

Table S4 Up-regulated differential expressed proteins of MeJA treatment for 24 h

| **NO.** | **GL-ID** | **Fold Change** | **Uniprot ID** | **Gene name** | **Protein description** |
| --- | --- | --- | --- | --- | --- |
| 1 | GL27042-R1_1 | 5.427 | P54781 | ERG5 | Cytochrome P450 61 |
| 2 | GL22068-R1_1 | 7.275 | P08524 | ERG20 | Farnesyl pyrophosphate synthase |
| 3 | GL24922-R1_1 | 1.694 | P54839 | ERG13 | Hydroxymethylglutaryl-CoA synthase |
| 4 | GL16081-R1_1 | 1.812 | Q03677 | ADI1 | 1,2-dihydroxy-3-keto-5-methylthiopentene dioxygenase |
| 5 | GL17912-R1_1 | 2.756 | P46367 | ALD4 | Potassium-activated aldehyde dehydrogenase |
| 6 | GL15986-R1_1 | 2.42 | P21595 | DIT2 | Cytochrome P450-DIT2 |
| 7 | GL27379-R1_1 | 2.245 | P38169 | BNA4 | Kynurenine 3-monooxygenase |
| 8 | GL28124-R1_1 | 3.04 | P23776 | EXG1 | Glucan 1,3-beta-glucosidase I/II |
| 9 | GL31571-R1_1 | 8.357 | Q04958 | NTE1 | Lysophospholipase |
| 10 | GL18309-R1_1 | 3.242 | P53037 | PSD2 | Phosphatidylserine decarboxylase proenzyme 2 |
| 11 | GL21327-R1_1 | 4.03 | P42884 | AAD14 | Putative aryl-alcohol dehydrogenase |
| 12 | GL24071-R2_1 | 9.439 | Q07747 | AAD4 | Probable aryl-alcohol dehydrogenase |
| 13 | GL18770-R1_1 | 5.245 | P50113 | LYS5 | phosphopantetheinyl transferase |
| 14 | GL19526-R1_1 | 2.052 | P39692 | MET10 | Sulfite reductase flavoprotein component |
| 15 | GL23738-R1_1 | 5.338 | P07806 | VAS1 | Valine--tRNA ligase |
| 16 | GL23263-R1_1 | 5.106 | Q05016 | YMR226C | NADP-dependent 3-hydroxy acid dehydrogenase |
| 17 | GL24163-R1_1 | 2.197 | P38113 | ADH5 | Alcohol dehydrogenase 5 |
| 18 | GL21539-R1_1 | 2.444 | P28240 | ICL1 | Isocitrate lyase |
| 19 | GL23892-R1_1 | 3.638 | P12866 | STE6 | Alpha-factor-transporting ATPase |
| 20 | GL30422-R1_1 | 3.604 | P40416 | ATM1 | Iron-sulfur clusters transporter |
| 21 | GL30705-R1_1 | 1.784 | P32568 | SNQ2 | Protein SNQ2 |
| 22 | GL26680-R1_1 | 99.083 | P39936 | TIF4632 | Eukaryotic initiation factor 4F subunit p130 |
| 23 | GL23833-R1_1 | 2.438 | Q03750 | TAF8 | Transcription initiation factor TFIID subunit 8 |
| 24 | GL18945-R1_1 | 4.447 | Q01939 | RPT6 | 26S proteasome regulatory subunit 8 homolog |
| 25 | GL20584-R1_1 | 6.037 | P33299 | RPT1 | 26S proteasome regulatory subunit 7 homolog |
| 26 | GL27501-R1_1 | 2.513 | P54867 | SLG1 | Protein SLG1 |
| 27 | GL21534-R1_1 | 2.637 | Q04195 | SIZ1 | E3 SUMO-protein ligase |
| 28 | GL25874-R1_1 | 8.316 | P53923 | NCS2 | Cytoplasmic tRNA 2-thiolation protein 2 |
| 29 | GL24602-R1_1 | 2.423 | P32795 | YME1 | i-AAA protease supercomplex subunit |
| 30 | GL31486-R1_1 | 2.534 | Q06628 | ATG13 | Autophagy-related protein 13 |
| 31 | GL20988-R1_1 | 3.294 | P38968 | SEC31 | Protein transport protein SEC31 |
| 32 | GL22536-R1_1 | 2.167 | P22023 | KRE5 | Killer toxin-resistance protein 5 |
| 33 | GL30115-R1_1 | 3.987 | P32906 | MNS1 | mannosyl-oligosaccharide 1,2-alpha-mannosidase |
| 34 | GL31667-R1_1 | 2.773 | Q00381 | APS2 | AP-2 complex subunit sigma |
| 35 | GL23031-R1_1 | 7.367 | P06700 | SIR2 | NAD-dependent histone deacetylase |
| 36 | GL15434-R1_1 | 4.32 | P38866 | FMO1 | Thiol-specific monooxygenase |
| 37 | GL16398-R1_1 | 5.202 | P38993 | FET3 | Iron transport multicopper oxidase |
| 38 | GL19367-R1_1 | 2.958 | P35190 | CLG1 | PHO85 cyclin CLG1 |
| 39 | GL19796-R1_1 | 2.088 | Q03921 | DOP1 | Protein dopey |
| 40 | GL19968-R1_1 | 23.496 | P22696 | ESS1 | Peptidyl-prolyl cis-trans isomerase |
| 41 | GL20414-R1_1 | 1.697 | P07278 | BCY1 | cAMP-dependent protein kinase regulatory subunit |
| 42 | GL20540-R1_1 | 6.494 | P29056 | BRF1 | Transcription factor IIIB 70 kDa subunit |
| 43 | GL20819-R1_1 | 2.529 | Q02207 | FOX2 | Peroxisomal hydratase-dehydrogenase-epimerase |
| 44 | GL21210-R1_1 | 2.109 | Q06010 | STE23 | A-factor-processing enzyme |
| 45 | GL21891-R1_1 | 2.739 | P02309 | HHF1 | Histone H4 |
| 46 | GL22213-R1_1 | 3.809 | P32901 | PTR2 | Peptide transporter |
| 47 | GL22721-R1_1 | 3.153 | P33400 | RIM101 | pH-response transcription factor pacC |
| 48 | GL23646-R1_1 | 1.73 | Q12482 | AGC1 | Mitochondrial aspartate-glutamate transporter |
| 49 | GL28634-R1_1 | 13.573 | P39937 | PAC2 | Protein PAC2 |
| 50 | GL29514-R1_1 | 2.629 | P53974 | ARK1 | Actin-regulating kinase 1 |
| 51 | GL29725-R1_1 | 7.867 | P50276 | MUP1 | High-affinity methionine permease |
| 52 | GL29873-R1_1 | 2.374 | P22146 | GAS1 | 1,3-beta-glucanosyltransferase |
| 53 | GL30376-R1_1 | 2.826 | Q08919 | TRE1 | Uncharacterized protein |
| 54 | GL31536-R1_1 | 1.7 | P53819 | YRF1-6 | helicase protein 1 copy 6 |
