# Supplementary material for: Integrated Proteomics and Metabolomics Analysis Provides Insights into Ganoderic Acid Biosynthesis in Response to Methyl Jasmonate in Ganoderma Lucidum
Source: Int J Mol Sci. 2019 Dec 4;20(24):6116. doi: 10.3390/ijms20246116 (PMC6941157; doi:10.3390/ijms20246116)
Supplement: Supplementary file 1 [file ijms-20-06116-s001.zip › ijms-642095-revised-r1-supplementary/Table S1-S12/Table S5 down M24.doc]

Table S5 Down-regulated differential expressed proteins of MeJA treatment for 24 h

| **NO.** | **GL-ID** | **Fold Change** | **Uniprot ID** | **Gene name** | **Protein description** |
| --- | --- | --- | --- | --- | --- |
| 1 | GL17001-R1_1 | 0.448 | P35497 | SOR1 | Sorbitol dehydrogenase 1 |
| 2 | GL25136-R1_1 | 0.55 | P14540 | FBA1 | Fructose-bisphosphate aldolase |
| 3 | GL22268-R1_1 | 0.514 | P00942 | TPI1 | Triosephosphate isomerase |
| 4 | GL20491-R1_1 | 0.421 | P04807 | HXK2 | Hexokinase-2（EMP） |
| 5 | GL22946-R1_1 | 0.487 | P53318 | COQ6 | Ubiquinone biosynthesis monooxygenase |
| 6 | GL24512-R1_1 | 0.563 | P53909 | AAH1 | Adenine deaminase |
| 7 | GL17501-R1_1 | 0.498 | P39954 | SAH1 | Adenosylhomocysteinase |
| 8 | GL31662-R1_1 | 0.34 | P05694 | MET6 | homocysteine methyltransferase |
| 9 | GL20474-R1_1 | 0.386 | Q04458 | HFD1 | Fatty aldehyde dehydrogenase |
| 10 | GL19766-R1_1 | 0.469 | P06208 | LEU4 | 2-isopropylmalate synthase |
| 11 | GL19372-R1_1 | 0.544 | P39522 | ILV3 | Dihydroxy-acid dehydratase |
| 12 | GL30076-R1_1 | 0.385 | P48570 | LYS20 | Homocitrate synthase, cytosolic isozyme |
| 13 | GL22111-R1_1 | 0.327 | P38999 | LYS9 | Saccharopine dehydrogenase |
| 14 | GL24538-R1_1 | 0.388 | P07702 | LYS2 | L-2-aminoadipate reductase |
| 15 | GL30526-R1_1 | 0.252 | P50264 | FMS1 | Polyamine oxidase |
| 16 | GL15299-R1_1 | 0.463 | P48016 | ATH1 | Vacuolar acid trehalase |
| 17 | GL31267-R1_1 | 0.427 | Q06625 | GDB1 | Glycogen debranching enzyme |
| 18 | GL25395-R1_1 | 0.299 | P23642 | VAN1 | Mannan polymerase I complex VAN1 subunit |
| 19 | GL29426-R1_1 | 0.596 | P41911 | GPD2 | Glycerol-3-phosphate dehydrogenase |
| 20 | GL26490-R1_1 | 0.232 | P32891 | DLD1 | D-lactate dehydrogenase 1 |
| 21 | GL21941-R1_1 | 0.292 | Q00955 | ACC1 | Acetyl-CoA carboxylase |
| 22 | GL25059-R1_1 | 0.302 | P52910 | ACS2 | Acetyl-coenzyme A synthetase 2 |
| 23 | GL16520-R1_1 | 0.212 | Q12068 | GRE2 | NADPH-dependent methylglyoxal reductase |
| 24 | GL21006-R1_1 | 0.429 | Q04792 | GAD1 | Glutamate decarboxylase |
| 25 | GL15281-R1_1 | 0.626 | P07284 | SES1 | Serine-tRNA ligase |
| 26 | GL21206-R1_1 | 0.511 | P04801 | THS1 | Threonine-tRNA ligase |
| 27 | GL25741-R1_1 | 0.425 | P09436 | ILS1 | Isoleucine-tRNA ligase |
| 28 | GL27820-R1_1 | 0.45 | P36421 | TYS1 | Tyrosine-tRNA ligase |
| 29 | GL30373-R1_1 | 0.537 | P07263 | HTS1 | Histidine-tRNA ligase |
| 30 | GL25227-R1_1 | 0.55 | P13188 | GLN4 | Glutamine-tRNA ligase |
| 31 | GL30558-R1_1 | 0.469 | P25358 | ELO2 | Elongation of fatty acids protein 2 |
| 32 | GL17430-R1_1 | 0.491 | P07259 | URA2 | Protein URA2 |
| 33 | GL31395-R1_1 | 0.317 | P07275 | PUT2 | Delta-1-pyrroline-5-carboxylate dehydrogenase |
| 34 | GL20810-R1_1 | 0.378 | P53954 | ALG11 | Mannosyltransferase |
| 35 | GL27363-R1_1 | 0.516 | P32190 | GUT1 | Glycerol kinase |
| 36 | GL31363-R1_1 | 0.514 | P14065 | GCY1 | Glycerol 2-dehydrogenase |
| 37 | GL30169-R1_1 | 0.248 | Q10740 | LAP2 | Leukotriene A-4 hydrolase homolog |
| 38 | GL24700-R1_1 | 0.423 | P53332 | CAB4 | Phosphopantetheine adenylyltransferase |
| 39 | GL24500-R1_1 | 0.517 | P46655 | GUS1 | Glutamate-tRNA ligase |
| 40 | GL31410-R1_1 | 0.173 | P21951 | POL2 | DNA polymerase epsilon catalytic subunit A |
| 41 | GL21375-R1_1 | 0.572 | P06738 | GPH1 | Glycogen phosphorylase |
| 42 | GL17081-R1_1 | 0.366 | P37292 | SHM1 | Serine hydroxymethyltransferase |
| 43 | GL29631-R1_1 | 0.519 | P22768 | ARG1 | Argininosuccinate synthase |
| 44 | GL19179-R1_1 | 0.44 | P18544 | ARG8 | Acetylornithine aminotransferase |
| 45 | GL29181-R1_1 | 0.459 | P32771 | SFA1 | S-(hydroxymethyl) glutathione dehydrogenase |
| 46 | GL22229-R1_1 | 0.578 | P00331 | ADH2 | Alcohol dehydrogenase 2 |
| 47 | GL30114-R1_1 | 0.411 | P00924 | ENO1 | Enolase 1 |
| 48 | GL24572-R1_1 | 0.47 | P06633 | HIS3 | Imidazoleglycerol-phosphate dehydratase |
| 49 | GL31286-R1_1 | 0.557 | P00815 | HIS4 | Histidine biosynthesis trifunctional protein |
| 50 | GL23873-R1_1 | 0.389 | P06169 | PDC1 | Pyruvate decarboxylase isozyme 1 |
| 51 | GL15683-R1_1 | 0.394 | P27616 | ADE1 | Succinocarboxamide synthase |
| 52 | GL16697-R1_1 | 0.577 | P38972 | ADE6 | Phosphoribosylformylglycinamidine synthase |
| 53 | GL30535-R1_1 | 0.419 | Q07505 | YDL086W | Putative carboxymethylenebutenolidase |
| 54 | GL19278-R1_1 | 0.395 | P07285 | TRP4 | Anthranilate phosphoribosyltransferase |
| 55 | GL20235-R1_1 | 0.318 | P00937 | TRP3 | Multifunctional tryptophan biosynthesis protein |
| 56 | GL29422-R1_1 | 0.332 | P40363 | YJL068C | S-formylglutathione hydrolase |
| 57 | GL24188-R1_1 | 0.505 | P19097 | FAS2 | Fatty acid synthase subunit alpha |
| 58 | GL19534-R1_1 | 0.543 | Q12196 | RIO1 | Serine/threonine-protein kinase |
| 59 | GL23666-R1_1 | 0.362 | P38882 | UTP9 | U3 small nucleolar RNA-associated protein 9 |
| 60 | GL22318-R2_1 | 0.449 | P0CX37 | RPS6A | 40S ribosomal protein S6-A |
| 61 | GL25744-R1_1 | 0.358 | P0CX56 | RPS18B | 40S ribosomal protein S18-B |
| 62 | GL28146-R1_1 | 0.534 | P07281 | RPS19B | 40S ribosomal protein S19-B |
| 63 | GL30395-R1_1 | 0.529 | P02407 | RPS17A | 40S ribosomal protein S17-A |
| 64 | GL31415-R1_1 | 0.312 | Q02326 | RPL6A | 60S ribosomal protein L6-A |
| 65 | GL31426-R1_1 | 0.461 | P26321 | RPL5 | 60S ribosomal protein L5 |
| 66 | GL19949-R1_1 | 0.419 | P46654 | RPS0B | 40S ribosomal protein S2-B |
| 67 | GL22820-R1_1 | 0.474 | P06103 | PRT1 | Eukaryotic translation initiation factor 3 subunit B |
| 68 | GL23081-R1_1 | 0.585 | P38249 | RPG1 | Eukaryotic translation initiation factor 3 subunit A |
| 69 | GL23794-R1_1 | 0.501 | P40217 | TIF34 | Eukaryotic translation initiation factor 3 subunit I |
| 70 | GL28710-R1_1 | 0.426 | P30771 | NAM7 | ATP-dependent helicase |
| 71 | GL24146-R1_1 | 0.583 | Q12099 | FAL1 | ATP-dependent RNA helicase |
| 72 | GL24973-R1_1 | 0.366 | P12385 | SUP45 | Eukaryotic peptide chain release factor subunit 1 |
| 73 | GL21067-R1_1 | 0.482 | P25644 | PAT1 | DNA topoisomerase 2-associated protein |
| 74 | GL21580-R1_1 | 0.619 | P0CS90 | SSC1 | Heat shock protein SSC1 |
| 75 | GL22152-R1_1 | 0.492 | Q12149 | RRP6 | Exosome complex exonuclease |
| 76 | GL31272-R1_1 | 0.305 | P43569 | CAF16 | CCR4-associated factor 16 |
| 77 | GL21804-R1_1 | 0.42 | P33203 | PRP40 | Pre-mRNA-processing protein |
| 78 | GL22604-R1_1 | 0.407 | P33334 | PRP8 | Pre-mRNA-splicing factor 8 |
| 79 | GL25322-R1_1 | 0.563 | Q12417 | PRP46 | Pre-mRNA-splicing factor |
| 80 | GL30704-R1_1 | 0.498 | P36048 | SNU114 | Pre-mRNA-splicing factor |
| 81 | GL31603-R1_1 | 0.332 | P40204 | SMX2 | Small nuclear ribonucleoprotein G |
| 82 | GL16506-R1_1 | 0.585 | P40016 | RPN3 | 26S proteasome regulatory subunit |
| 83 | GL24074-R1_1 | 0.474 | P43588 | RPN11 | Ubiquitin carboxyl-terminal hydrolase |
| 84 | GL18548-R1_1 | 0.437 | P48562 | CLA4 | Serine/threonine-protein kinase |
| 85 | GL29836-R2_1 | 0.458 | P39960 | BEM2 | GTPase-activating protein BEM2/IPL2 |
| 86 | GL22189-R1_1 | 0.313 | P06115 | CTT1 | Catalase T |
| 87 | GL31145-R1_1 | 0.431 | P24869 | CLB2 | G2/mitotic-specific cyclin-2 |
| 88 | GL20605-R1_1 | 0.334 | P32491 | MKK2 | MAP kinase kinase MKK2/SSP33 |
| 89 | GL24589-R1_1 | 0.357 | P14681 | KSS1 | Mitogen-activated protein kinase |
| 90 | GL31443-R1_1 | 0.337 | P17442 | PHO81 | Phosphate system positive regulatory protein |
| 91 | GL28407-R1_1 | 0.361 | P08678 | CYR1 | Adenylate cyclase |
| 92 | GL20160-R1_1 | 0.462 | Q03280 | TOM1 | E3 ubiquitin-protein ligase |
| 93 | GL21841-R1_1 | 0.496 | P52492 | UBC11 | Ubiquitin-conjugating enzyme E2 |
| 94 | GL31556-R1_1 | 0.481 | P22515 | UBA1 | Ubiquitin-activating enzyme E1 1 |
| 95 | GL30014-R1_1 | 0.329 | P53886 | APC1 | Anaphase-promoting complex subunit 1 |
| 96 | GL30164-R1_1 | 0.529 | Q12440 | APC2 | Anaphase-promoting complex subunit 2 |
| 97 | GL22277-R1_1 | 0.398 | P54861 | DNM1 | Dynamin-related protein |
| 98 | GL23367-R1_1 | 0.329 | P15801 | MIP1 | DNA polymerase gamma |
| 99 | GL24667-R1_1 | 0.318 | P15108 | HSC82 | ATP-dependent molecular chaperone |
| 100 | GL21736-R1_1 | 0.534 | P25694 | CDC48 | Cell division control protein 48 |
| 101 | GL24777-R1_1 | 0.409 | Q08109 | HRD1 | ERAD-associated E3 ubiquitin-protein ligase |
| 102 | GL24688-R1_1 | 0.414 | P10591 | SSA1 | Heat shock protein |
| 103 | GL30233-R1_1 | 0.586 | P22202 | SSA4 | Heat shock protein |
| 104 | GL26617-R1_1 | 0.515 | P19146 | ARF2 | ADP-ribosylation factor 2 |
| 105 | GL25065-R1_1 | 0.535 | P32573 | SPS19 | Peroxisomal 2,4-dienoyl-CoA reductase |
| 106 | GL18900-R1_1 | 0.401 | P14742 | GFA1 | Glutamine-fructose-6-phosphate aminotransferase |
| 107 | GL25257-R1_1 | 0.527 | P32598 | GLC7 | Serine/threonine-protein phosphatase PP1-2 |
| 108 | GL17558-R1_1 | 0.525 | P38219 | OLA1 | Obg-like ATPase 1 |
| 109 | GL18176-R1_1 | 0.628 | P53970 | EFM6 | Protein-lysine N-methyltransferase |
| 110 | GL19075-R1_1 | 0.309 | P00431 | CCP1 | Cytochrome c peroxidase |
| 111 | GL19388-R1_1 | 0.346 | Q08240 | YOL099C | Putative uncharacterized membrane protein |
| 112 | GL20609-R1_1 | 0.56 | P38723 | COS8 | Protein COS8 |
| 113 | GL20673-R1_1 | 0.276 | P38872 | PFS1 | Prospore formation at selected spindle poles protein 1 |
| 114 | GL20744-R1_1 | 0.477 | P32454 | APE2 | Aminopeptidase 2 |
| 115 | GL21274-R1_1 | 0.549 | P21339 | MSB1 | Morphogenesis-related protein |
| 116 | GL21535-R1_1 | 0.537 | P35208 | SPT10 | Protein SPT10 |
| 117 | GL21604-R1_1 | 0.589 | P25386 | USO1 | Intracellular protein transport protein |
| 118 | GL21796-R1_1 | 0.459 | P27614 | CPS1 | Carboxypeptidase S |
| 119 | GL22129-R1_1 | 0.468 | P31115 | DEG1 | tRNA pseudouridine(38/39) synthase |
| 120 | GL22582-R1_1 | 0.4 | P38149 | DUG2 | Probable di- and tripeptidase |
| 121 | GL22889-R1_1 | 0.459 | P53388 | DIP5 | Dicarboxylic amino acid permease |
| 122 | GL22951-R1_1 | 0.612 | P30619 | SEC1 | Protein transport protein |
| 123 | GL23350-R1_1 | 0.637 | Q08930 | YPL191C | Ubiquitin carboxyl-terminal hydrolase |
| 124 | GL23547-R2_1 | 0.396 | Q03048 | COF1 | Cofilin |
| 125 | GL23624-R1_1 | 0.263 | Q12180 | HAL9 | Halotolerance protein 9 |
| 126 | GL23700-R1_1 | 0.324 | P29547 | CAM1 | Elongation factor 1-gamma 1 |
| 127 | GL23744-R1_1 | 0.509 | P46672 | ARC1 | tRNA-aminoacylation cofactor |
| 128 | GL23804-R1_1 | 0.479 | P40037 | HMF1 | Protein HMF1 |
| 129 | GL24313-R1_1 | 0.415 | P40083 | YER137C | Uncharacterized protein |
| 130 | GL24443-R1_1 | 0.515 | P33892 | GCN1 | eIF-2-alpha kinase activator |
| 131 | GL24645-R1_1 | 0.449 | Q07904 | THI73 | Thiamine pathway transporter |
| 132 | GL24774-R1_1 | 0.458 | P53912 | YNL134C | Uncharacterized protein YNL134C |
| 133 | GL24836-R1_1 | 0.578 | P50101 | UBP15 | Ubiquitin carboxyl-terminal hydrolase 15 |
| 134 | GL25411-R1_1 | 0.349 | P04786 | TOP1 | DNA topoisomerase 1 |
| 135 | GL25987-R1_1 | 0.288 | P38174 | MAP2 | Methionine aminopeptidase 2 |
| 136 | GL26084-R1_1 | 0.215 | P46682 | APL6 | AP-3 complex subunit beta |
| 137 | GL26098-R1_1 | 0.515 | P33313 | CNS1 | Hsp70/Hsp90 co-chaperone |
| 138 | GL26482-R1_1 | 0.539 | P38699 | STB5 | Protein STB5 |
| 139 | GL26639-R1_1 | 0.428 | P20449 | DBP5 | ATP-dependent RNA helicase |
| 140 | GL27497-R1_1 | 0.418 | P38851 | YSP1 | Membrane-anchored lipid-binding protein |
| 141 | GL28199-R1_1 | 0.618 | P40309 | KHA1 | K(+)/H(+) antiporter 1 |
| 142 | GL28218-R1_1 | 0.344 | P25375 | PRD1 | Saccharolysin |
| 143 | GL30522-R1_1 | 0.4 | P38237 | UBP14 | Ubiquitin carboxyl-terminal hydrolase 14 |
| 144 | GL30543-R1_1 | 0.373 | P40093 | YER156C | UPF0160 protein |
| 145 | GL31094-R1_1 | 0.414 | Q01476 | UBP2 | Ubiquitin carboxyl-terminal hydrolase 2 |
| 146 | GL31173-R1_1 | 0.262 | Q02799 | LEE1 | Zinc finger protein |
| 147 | GL31548-R1_1 | 0.384 | Q08225 | YOL057W | Probable dipeptidyl peptidase 3 |
| 148 | GL24736-R1_1 | 0.461 | P47028 | YJL086C | Putative uncharacterized protein |
